# Asymmetric generalizability of multimodal brain‐behavior associations across age‐groups

**DOI:** 10.1002/hbm.26035

**Published:** 2022-07-30

**Authors:** Junhong Yu, Nastassja L. Fischer

**Affiliations:** ^1^ Psychology, School of Social Sciences National Technological University Singapore Singapore; ^2^ Centre for Research and Development in Learning (CRADLE) Nanyang Technological University Singapore Singapore

**Keywords:** aging, agegeneralizability, gray matter, resting state functional connectivity, structural connectivity

## Abstract

Machine learning methods have increasingly been used to map out brain‐behavior associations (BBA), and to predict out‐of‐scanner behavior of unseen subjects. Given the brain changes that occur in the context of aging, the accuracy of these predictions is likely to depend on how similar the training and testing data sets are in terms of age. To this end, we examined how well BBAs derived from an age‐group generalize to other age‐groups. We partitioned the CAM‐CAN data set (*N* = 550) into the young, middle, and old age‐groups, then used the young and old age‐groups to construct prediction models for 11 behavioral outcomes using multimodal neuroimaging features (i.e., structural and resting‐state functional connectivity, and gray matter volume/cortical thickness). These models were then applied to all three age‐groups to predict their behavioral scores. When the young‐derived models were used, a graded pattern of age‐generalization was generally observed across most behavioral outcomes—predictions are the most accurate in the young subjects in the testing data set, followed by the middle and then old‐aged subjects. Conversely, when the old‐derived models were used, the disparity in the predictive accuracy across age‐groups was mostly negligible. These findings hold across different imaging modalities. These results suggest the asymmetric age‐generalization of BBAs—old‐derived BBAs generalized well to all age‐groups, however young‐derived BBAs generalized poorly beyond their own age‐group.

## Graphical abstract

We examined how well brain‐behavior associations derived from an age‐group generalize to other age‐groups. Our results showed that these associations derived from an old‐aged neuroimaging data set generalized well to the young individuals; however, the reverse is not true—Young‐derived brain‐behavior associations generalized poorly to older adults.

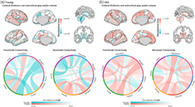

## 1 INTRODUCTION

One of the major goals of neuroimaging research is to identify associations between brain‐related characteristics and behavioral functions and potentially use this information to predict an individual's out‐of‐scanner behavior. Generally, such research would first model the associations between a behavioral measure and multivariate neuroimaging features—typically resting‐state functional connectomes (rsFC), within a training data set. Subsequently, this model is applied to the neuroimaging features of an unseen data set to predict the unseen subjects' behavioral outcomes.

While it is largely conceivable that the accuracy of these predictions would depend on the similarity between the train and test data sets, this hypothesis has not been adequately explored in the literature. Importantly, this “similarity” can be conceptualized in terms of age, and, therefore, when exploring its impact on the accuracy of a model's prediction, one should consider the brain's potential changes throughout development.

When accounting for the individual differences in brain aging, previous work has attributed these to the likely interactive effects of three main mechanisms: reserve, maintenance, and compensation (Knyazev et al., 2015). In this way, across the lifespan, the human brain would undergo extensive changes as a result of attaining various developmental milestones, neuroplasticity and age‐related atrophy of brain structures (Battaglini et al., 2019), as well as age‐related reorganization (Stumme et al., 2020) and dedifferentiation (Koen et al., 2020) of brain networks. However, while these processes may be considered as adaptive mechanisms for neural resilience and can be indicative of a healthy aging process (Chan et al., 2014), variations in the brain's connectivity arrangements are believed to be a consequence of differences in information processing between specialized brain regions (Salat et al., 2005). Consequently, the brain mechanisms underpinning a behavioral function may change across aging. Thus, while a set of neuroimaging features may be predictive of a behavioral measure for a certain age‐group, it may not be so for another age‐group.

Findings on the generalizability of BBAs across age‐group have been scant and mixed. One study (Gao et al., 2020) modeled the associations between rsFC and processing speed in an old‐age sample and found that this model predicted processing speed scores poorly in unseen young and middle‐age subjects as compared to unseen old‐age subjects. As expected, this preliminary finding suggested that BBAs are age‐specific, they did not generalize well beyond the age‐group they were derived from. A more recent study (Yu & Fischer, 2022) modeled the associations between brain connectomes—both resting‐state functional and structural connectomes, and a diverse spectrum of behavioral outcomes in a young sample, and showed that when these BBAs are applied to an unseen young and old‐age samples, a similar age‐specificity effect was observed—behavioral outcomes were predicted more accurately in the unseen young than old age‐groups. Furthermore, among the unseen old subjects, cognitive functions were predicted poorly as compared to behavioral outcomes in other domains such as personality and affect. This suggested that the age‐generalizability of BBAs varies across behavioral domains, in particular, BBAs in the cognitive domain were the least age‐generalizable. However, these studies were largely limited by the fact that they had only examined young‐to‐old or old‐to‐young generalizability, but not generalizability in both directions. It is tempting to assume that the degree of young‐to‐old and old‐to‐young generalizability of BBAs are similar, however, this assumption has yet to be tested empirically.

In the current study, we examined how the generalizability of multimodal BBA from 11 behavioral/cognitive outcomes varies across age‐groups. Specifically, we hypothesized that age‐generalizability effects would vary in a graded manner across the age spectrum. Hence, the greater the age differences between the train and test samples, the poorer the BBAs derived from the training data set would generalize to the test sample. Additionally, we examined the age‐generalizability of BBAs in both directions (i.e., young‐to‐old and old‐to‐young); and separately used the young and old age‐groups as the training samples to examine their generalizability to other age‐groups. We hypothesized that the generalizability of BBAs from young‐to‐old and old‐to‐young are similar.

## 2 METHODS

### 2.1 Participants

**FIGURE 1:**
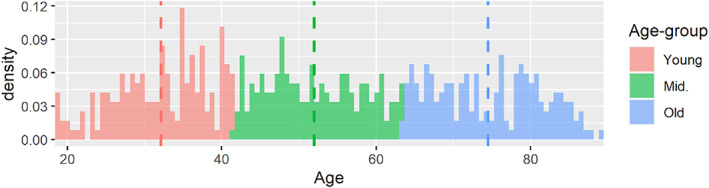
Density plot illustrating the age distribution in the three age groups. The dashed lines represent the mean age of their respective age‐groups

Data used in the preparation of this work were obtained from the CamCAN repository (available at http://www.mrc-cbu.cam.ac.uk/datasets/camcan/). Specifically, we used data acquired from stage 2 of the CamCAN study, which included 656 participants. Information regarding the recruitment procedures and exclusion criteria have been described in detail elsewhere (Shafto et al., 2014; Taylor et al., 2017). After excluding subjects with missing MRI scans and those with excessive head motion during their rsfMRI scans, the final studied sample consisted of 550 subjects. They were then partitioned into three approximately equal groups (*N* = 183, 184, and 183) according to their age. Figure 1 illustrates these partitioned age groups. These age groups are subsequently referred to as the “young” (18.5 ≤ age ≤ 41.5), “middle” (41.7 ≤ age ≤ 63.5), and “old” (63.6 ≤ age ≤ 88.9). The proportions of females in these three age groups are similar (53.6%, 45.7%, and 49.2%). The CamCAN study was conducted in compliance with the Helsinki Declaration and has been approved by the local ethics committee, Cambridgeshire 2 Research Ethics Committee (reference: 10/H0308/50).

### 2.2 Behavioral measures

The CAM‐CAN data set included 14 behavioral tasks which were administered outside of the scanner and a sensorimotor task that was carried out during an fMRI scan. For each of these tasks, several behavioral outcomes were measured. As it would not be possible to study all of them in the current study. We only included them in the study if they were identified as “Key Variables” in the protocol paper (Taylor et al., 2017) and less than 10% of the data are missing or invalid. Additionally, some behavioral tasks recorded error scores that were mostly “0s” and these were excluded from our study. A total of 11 behavioral outcomes from 9 tasks were included. The description of these tasks and outcomes is shown in Table 1.

**TABLE 1:** Description of behavioral tasks and measures included in the study

Task	Description	Outcomes
Emotion expression recognition	Subject viewed faces and identify their expressed emotions (happy, sad, anger, fear, disgust, surprise).	• Average accuracy across all types of emotion expressions
Face recognition: familiar faces	Subject viewed faces of famous people and unknown foils and determine they are familiar. If they are deemed to be familiar, the subject will proceed to identify the person's name and occupation.	• Number of famous faces named • Number of famous occupations given
Face recognition: unfamiliar faces	Subject is shown a target person's face, and was asked to identify this person in an array of 6 face images.	• Accuracy
Cattell culture fair intelligence test, Scale 2	Subject completed 4 subtests, which involved completing a sequence of drawings, identifying a drawing that is different from others, completing a matrix of patterns, and identifying a geometric design among several others that fulfills a specific given condition.	• Total score across 4 subtests
Hotel task	Subject will take role of a hotel manager to perform some tasks, such as writing customer bills, sorting money, proofreading ads, sorting playing cards, arranging list of names alphabetically. There is not enough time to complete any single task and time must be allocated equally between tasks.	• Number of tasks attempted • Deviation from optimal time allocation
RT choice	Subject viewed an image of a hand with blank circles above the fingers, while resting their right hand on a response box with four buttons, one for each finger. On each trial, any one of the four circles above the fingers in the image could become black, and the subject must press the corresponding finger as quickly as possible. Each trial is separated from the next with a variable inter‐trial interval (blank screen).	• Mean response time
Proverbs comprehension	Subject read and interpreted three English proverbs.	• Total score
Sensorimotor task	Subject pressed a button when they see two circular checkerboards visually presented simultaneously to the left and right of a central fixation cross and/or hear a binaural tone. These task trials were combined with null trials of the same length.	• Mean response time
Visual short‐term memory	Subject is presented with one to four colored discs on a computer screen for a brief duration. After a delay, the subject will indicate the color of the discs that had appeared at a cued location.	• Average accuracy of reported color across all 4 set sizes

### 2.3 MRI acquisition

Participants were scanned using a 3T Siemens TIM Trio scanner equipped with a 32‐channel head coil. T1‐weighted images were acquired using a MPRAGE protocol (TE = 2.99 ms; TR = 2250 ms; FOV = 256 × 240 × 192 mm; voxel size = 1 mm isotropic). For the resting‐state fMRI, 261 EPI volumes were acquired (TR = 1970 ms; TE = 30 ms; 32 axial slices; FOV = 192 × 192; voxel size = 3 × 3 × 4.44 mm). During the resting‐state fMRI scan, subjects were instructed to remain awake and lie still with their eyes open while looking at a fixation cross. Diffusion‐weighted images were acquired using a Twice‐refocused spin‐echo sequence, consisting of 3 *b*_0_ images, 30 diffusion directions at *b* = 1000 s/mm^2^ and *b* = 2000 s/mm^2^ (TE = 104 ms; TR = 9100 ms; FOV = 192 × 192, 66 axial slices, voxel size = 2 mm isotropic). Additionally, gradient echo field maps (TR = 400 ms; TE1 = 5.19 ms; TE2 = 7.65 ms) were acquired for correcting the rsfMRI distortions.

### 2.4 Image processing

The T1 structural images were preprocessed with FreeSurfer 7.2.0 using the default recon‐all options. Briefly, this processing included the removal of non‐brain tissue using a hybrid watershed/surface deformation procedure (Segonne et al., 2004), automated Talairach transformation, segmentation of the subcortical white matter and deep gray matter volumetric structures (including hippocampus, amygdala, caudate, putamen, ventricles) (Fischl et al., 2002, 2004) intensity normalization (Sled et al., 1998), tessellation of the gray matter white matter boundary, automated topology correction (Fischl et al., 2001; Segonne et al., 2007), and surface deformation following intensity gradients to optimally place the gray/white and gray/cerebrospinal fluid borders at the location where the greatest shift in intensity defined the transition to the other tissue class (Dale et al., 1999; Fischl & Dale, 2000). Once the cortical models were complete, they were registered to a spherical atlas which was based on individual cortical folding patterns to match cortical geometry across subjects (Fischl et al., 1999), and the cerebral cortex is parcellated into units with respect to gyral and sulcal structure. This method used both intensity and continuity information from the entire three‐dimensional MR volume in segmentation and deformation procedures to produce representations of cortical thickness, calculated as the closest distance from the gray/white boundary to the gray/CSF boundary at each vertex on the tessellated surface (Fischl & Dale, 2000). Mean cortical thickness (CT) in mm and subcortical volumes in mm^3^ were extracted via the Destrieux (Destrieux et al., 2010) and Aseg (Fischl et al., 2002) atlases, respectively. Then, the subcortical volumetric measurements for each subject were divided by the subject's estimated total intracranial volume. These TIV‐corrected subcortical measurements were then combined with CT measurements, across all subjects, into a single matrix which was used as input features in the subsequent analyses.

The preprocessing of the diffusion images, tractography, and construction of the structural connectome were carried out using MRtrix3 (version 3.0.3) (Tournier et al., 2019). Briefly, MP‐PCA denoising (Veraart et al., 2016) and removal of Gibbs ringing artifacts (Kellner et al., 2016) were carried out on the raw images. Subsequently, they were corrected for motion, eddy currents and susceptibility‐induced distortion using the inhomogeneity field maps obtained previously. Following this, bias field correction (Tustison et al., 2010) was carried out. Next, the gray matter (GM), white matter (WM), and cerebrospinal fluid (CSF) response functions were obtained using the Dhollander (Dhollander & Connelly, 2016) algorithm which were in turn used for estimating fiber orientation distributions (FOD) in the WM, GM and CSF tissues from diffusion data using spherical deconvolution. Then, anatomically constrained tractography (Smith et al., 2012) was carried out using the WM FOD. This involved the prior preparation of a GM mask from the Freesurfer segmentations and then using this mask to seed streamlines.

The AAL‐90 atlas (Tzourio‐Mazoyer et al., 2002) was used to parcellate the whole brain. For each subject, the AAL‐90 template was first warped to the subject's native DTI space to obtain the transformations. Then, the warped template was overlaid onto the subject's diffusion tensors for visual inspection of the alignment. The transformations obtained previously were then applied to warp the AAL‐90 atlas into the subject's native diffusion space. For the network construction in the subject's native diffusion space, the nodes *i* and *j* were thought to be connected by an edge (*e*_*ij*_ = [*i*, *j*]), if at least one reconstructed streamline was found with its two endpoints located within the two nodes, respectively. The edges in the connectivity matrix for each participant were operationalized as the number of streamlines connecting between each pair of regions. Finally, the thresholded matrices were normalized using the Brain Connectivity Toolbox (Rubinov & Sporns, 2010) within MATLAB.

Using FSL FUGUE, the gradient echo field maps were applied to the resting‐state fMRI volumes to correct for distortions due to magnetic field inhomogeneities. These distortion‐corrected volumes were then subsequently preprocessed using fMRIPrep 20.2.5 (Esteban et al., 2019). Functional data were slice time corrected using 3dTshift from AFNI (Cox, 1996) and motion‐corrected using MCFLIRT (Jenkinson et al., 2002). This process was followed by co‐registration to the corresponding T1w using boundary‐based registration (Greve & Fischl, 2009) with 9 degrees of freedom, using bbregister from freesurfer. Motion correcting transformations, BOLD‐to‐T1w transformation and T1w‐to‐template (MNI) warp were concatenated and applied in a single step using antsApplyTransforms employing Lanczos interpolation.

Subsequently, these preprocessed volumes were denoised by regressing out 6 motion parameters, the average signal of white matter and cerebrospinal fluid masks, global signal, and their derivatives, as well as cosines covering slow time drift frequency band using the load_confounds package (https://github.com/SIMEXP/load_confounds) in Python. Scrubbing was carried out using the Power et al. (2014) approach to further remove the effects of excessive head motion. The volumes were then smoothed using a 5 mm FWHM kernel and subjected to a 0.1 Hz low‐pass filter. Finally, the brainnetome atlas (Fan et al., 2016) was used to parcellate the whole brain into 246 anatomical regions corresponding to the nodes of the network. Participants with excessive head motion (root mean squared displacement >0.25) were excluded from the analyses.

The edge values in the upper triangle of the SC and rsFC connectivity matrices were vectorized and concatenated across subjects. The vectorized SC and rsFC matrices were then used as input features in the subsequent analyses.

### 2.5 Statistical analysis

Ridge regression (RR) models were used separately on the three modalities (GM, SC, rsFC) to construct prediction models for each behavioral measure within an age group, which were subsequently applied to the other age groups. RR is intended for multiple regression scenarios in which the predictors are highly correlated with each other, such as in the case of our neuroimaging predictors. Unlike ordinary least squares regression, RR imposes an L^2^‐norm penalty on its regression coefficients to minimize the residual sum of squares.

First, the participants in the young sample were randomly assigned to a training (young‐train) and testing data set (young‐test) using an approximate 3:1 train‐test split. In order to minimize overfitting, the optimal tuning parameter (*λ*) was determined via an internal 5‐fold cross‐validation (CV) procedure within the young‐train sample. The range of *λ* values tested was automatically determined using the R package “glmnet.” Across this range of *λ* values, we selected the largest *λ* value that has a mean squared error (MSE) within one standard error of the minimum MSE (Gao et al., 2019) in the 5‐fold CV. These tuned RR models, derived from the young‐train data set, are then applied to the young‐test, middle and old data sets to generate behavioral predictions for each age group in the three modalities.

Additionally, we also generated prediction models using a fourth “modality” (i.e., stacked modality) that stacks the three modalities together using a random forest (RF) model, as implemented in the R package “caret.” In this RF model, the RR predicted scores for each of the three modalities were used as input features to predict behavioral scores within the young‐train sample. The optimal “mtry” parameter was tuned using a 5‐fold CV in a similar manner. This tuned RF model was then applied to the young‐test, middle‐ and old‐age data sets to generate behavioral predictions for each age group in the stacked modality.

To assess the accuracy of predictions in the young‐test data set we computed the normalized root mean square error (NRMSE) between the predicted and observed scores for all four modalities. The NRMSE is the root mean square error (RMSE) divided by the mean observed score. The NRMSE can be compared across measures and samples; lower NRMSE values correspond to more accurate predictions. To assess the degree of age generalizability, we calculate the ratios of RMSE_young_/RMSE_old_ and RMSE_young_/RMSE_middle_. These RMSE ratios can similarly be compared across measures. Ratios close to 1 meant that the model predicted scores at similar levels of precision in both the young‐test and old/middle samples, alluding to greater generalizability of the young‐train model to the old/middle samples. On the other hand, RMSE ratios below 1 indicate that predictions were more accurate in the young‐test than in the old/middle samples, and smaller ratios suggest greater discrepancies in the accuracies between the young test and old/middle samples. We opted not to use the correlation between predicted and actual scores (i.e., *r*) and percentage variance explained (i.e., *R* ^2^), because these metrics depends on the relationship between the predicted and actual scores and not so much the absolute precision of the predictions. It is possible to obtain a high *r* or *R* ^2^ even if there are major discrepancies between the predicted and actual scores, if these discrepancies are relatively consistent across all subjects (i.e., affine transformation). Additionally, these metrics also depend on the variability in scores which can be a problem in small test samples, such as in the current scenario where the young sample was partitioned using a 3:1 train‐test ratio.

Finally, we also calculated the predictor importance of the three modalities from the RF model and took their ratios as an approximate indicator of their share of contribution in predicting the behavioral scores in the young‐train sample.

**FIGURE 2:**
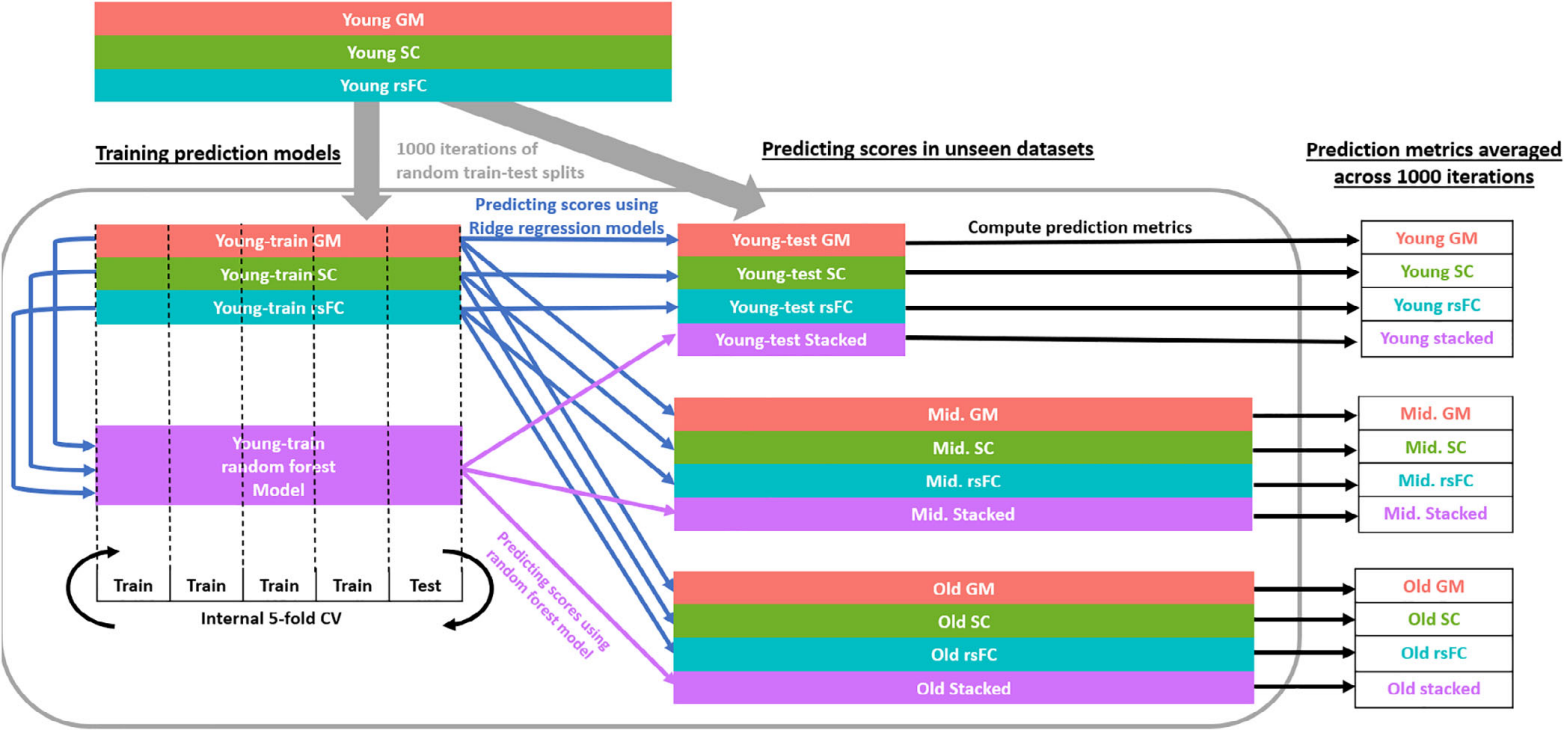
An overview of the train‐test procedures. In this instance, the young age group was used as the train sample to examine age‐generalizability in the middle and old age groups. These procedures are subsequently repeated with the old age group as the train sample, to examine age‐generalizability in the young and middle age groups. CV, cross‐validation; GM, gray matter; rsFC, resting‐state functional connectivity; SC, structural connectivity

Given that the way the young subjects were shuffled into the young‐train and young‐test samples would affect the optimal *λ* value and subsequently the beta coefficients and prediction metrics, the above‐mentioned procedures were repeated 1000 times, with participants randomly assigned into the young‐train and young‐test samples each time. After which, the prediction metrics and beta coefficients obtained at the end of each iteration were averaged across the 1000 iterations. An overview of these train‐test procedures is illustrated in Figure 2.

Finally, these procedures were repeated using the old age group as the training sample. That is, the old age group was similarly partitioned into old‐train and old‐test data sets. RR and RF models were trained in the old‐train data set which were then applied to the old‐test, young and middle‐age samples.

For the comparison of the model characteristics and prediction metrics between modalities or age groups, we used paired samples *t*‐tests and repeated measures analysis of variance (ANOVA) with the age‐group as the within factor. Statistical significance was set at *p* < .05. All analyses are carried out in R 4.0.2. The R codes for the analyses and generating the figures, together with the preprocessed data analyzed in this study are available at https://osf.io/wbhkg/.

## 3 RESULTS

### 3.1 Model characteristics

**FIGURE 3:**
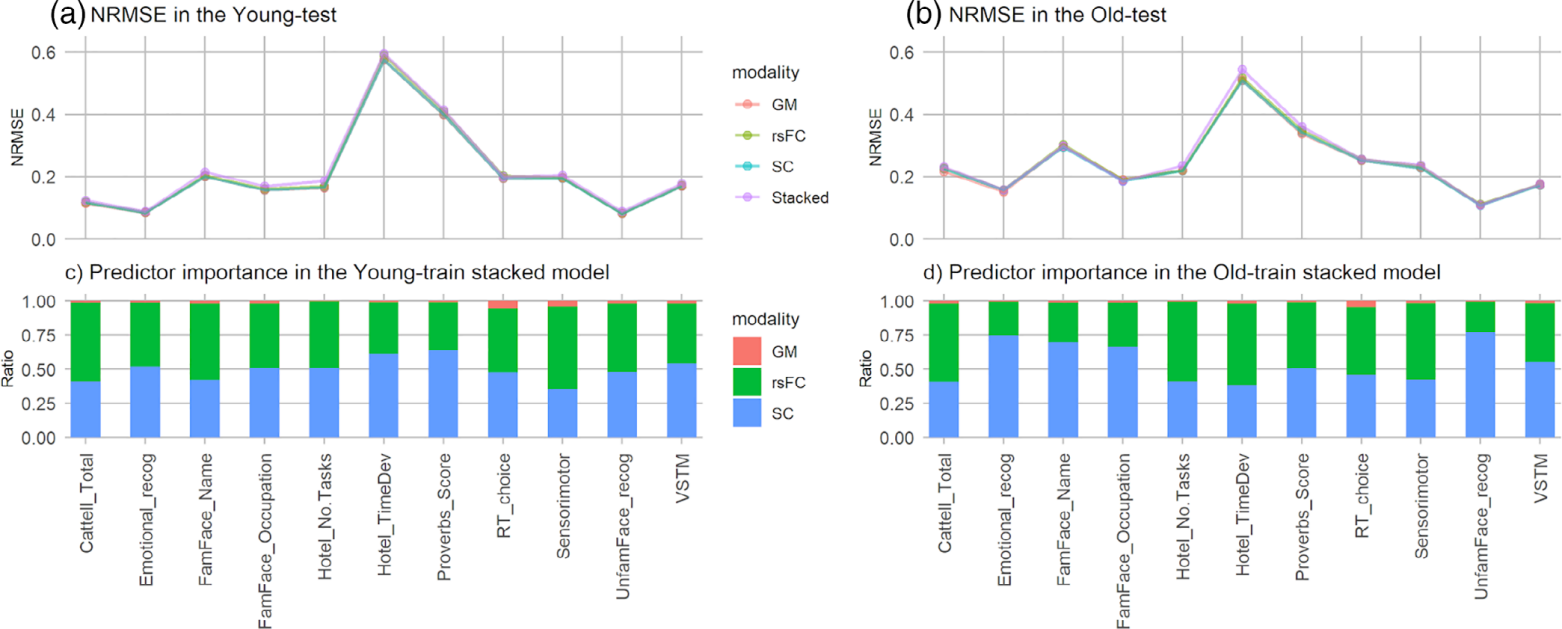
Predictive accuracy (a and b) and importance (c and d) of the different modalities in the young and old age‐groups, across all 11 behavioral measures. GM, gray matter; NRMSE, normalized root mean square error; rsFC, resting‐state functional connectivity; SC, structural connectivity

**FIGURE 4:**
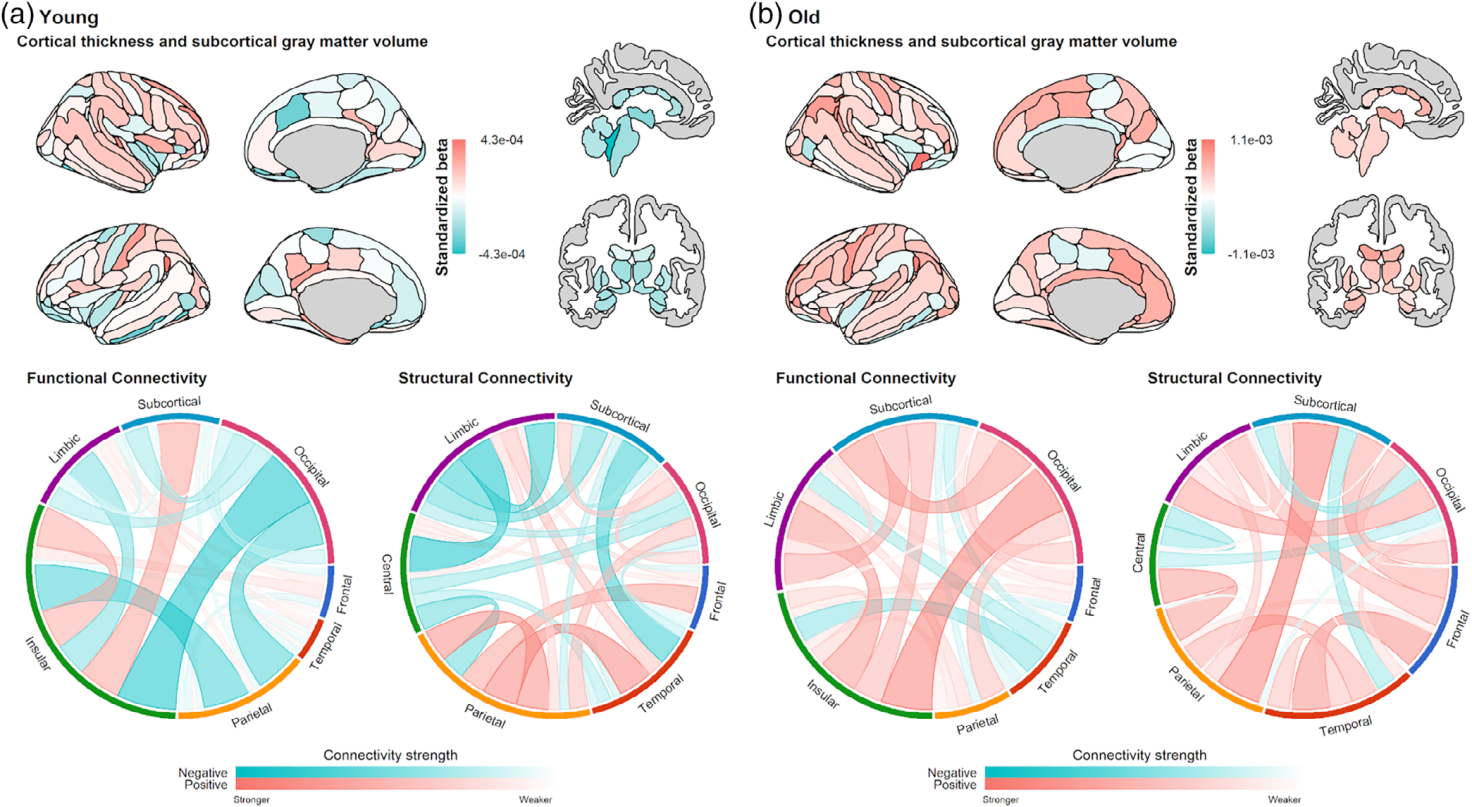
Beta coefficients predicting “UnfamFace_Recog” scores which were obtained in the gray matter, resting‐state functional connectivity, and structural connectivity modalities, averaged across 1000 permuted train‐test iterations in the (a) young and (b) old training samples. For the purpose of illustrating the region‐wise resting‐state functional and structural connectivity profiles in the chord diagrams, the edge beta coefficients are averaged across their respective regions. Details regarding the mapping of the nodes to their respective brain regions in the functional and structural connectomes are available at https://atlas.brainnetome.org/bnatlas.html and from Table 3 of Rolls et al. (2015), respectively

Four sets of prediction models, each corresponding to a modality were trained in the young and old‐age groups. The prediction performance of these models in an unseen test data set of a similar age group is illustrated in Figure 3a,b. In general, differences in NRMSE between the modalities were negligible (*p*s ≥ .996; partial *η* ^2^ ≤ .0015) as compared to the differences across behavioral measures. The ratio of predictor importance values suggested that the SC and rsFC modalities contributed mostly to the stacked models' predictions in both the young‐train and old‐train data sets. In both the young‐test and old‐test data sets, these prediction models performed the best on “UnfamFace_Recog” and the worst on “Hotel_No.Tasks.” An example of the beta coefficients obtained in the RR models, such as in the case of “UnfamFace_Recog” within the young‐train and old‐train samples, is illustrated in Figure 4. Similar plots for other behavioral outcomes are presented in the supplementary materials.

### 3.2 Age generalizability

**FIGURE 5:**
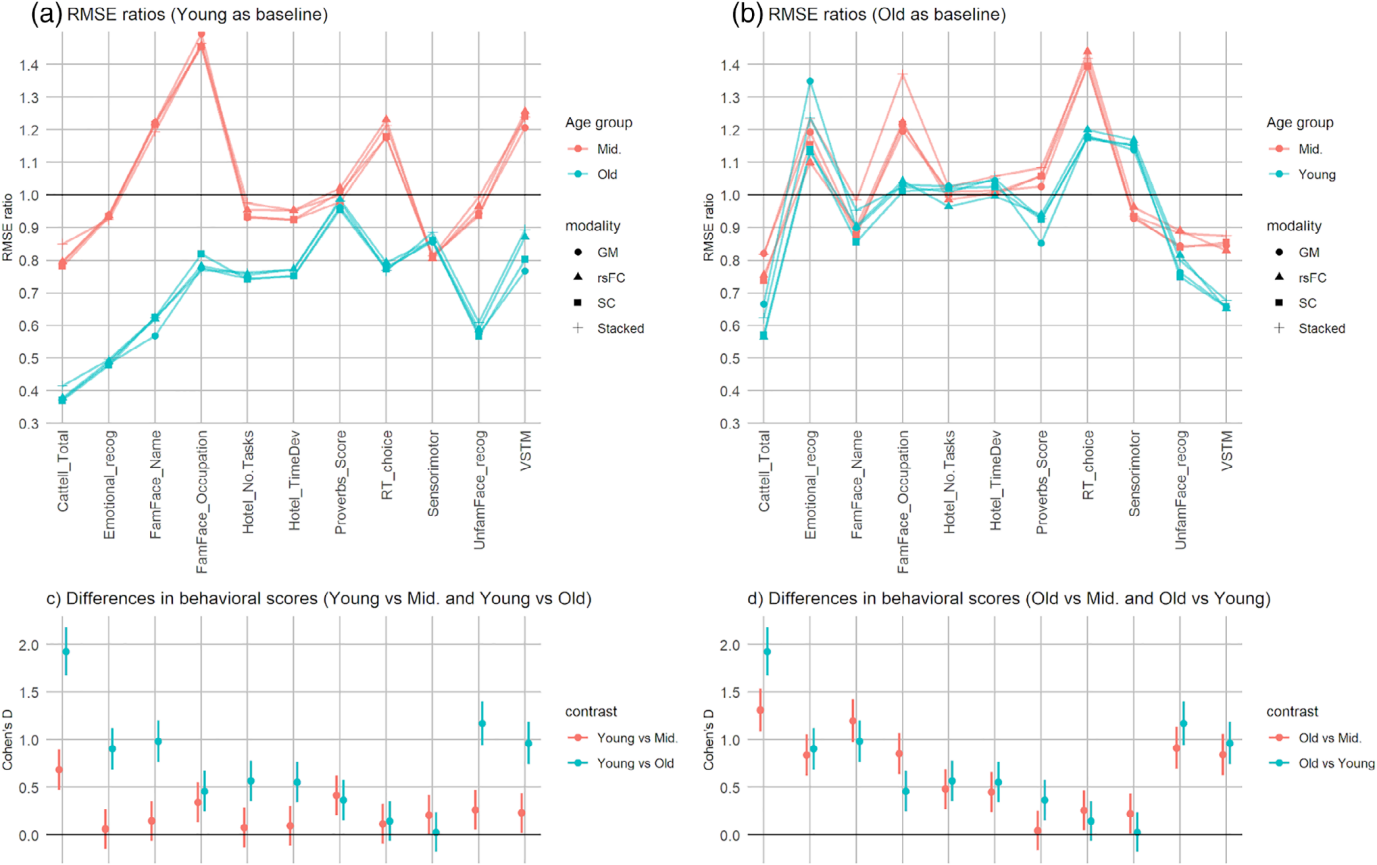
RMSE ratios illustrating the (a) young‐to‐middle, young‐to‐old, (b) old‐to‐middle, and old‐to‐young age‐generalizability of multimodal brain‐behavior associations. Higher RMSE ratios correspond to greater generalizability. In order to illustrate the relationship between age differences in behavioral scores and age‐generalizability of brain‐behavior associations, the Cohen's *D*s of the relevant age‐group contrasts are displayed in the bottom panel (c) and (d). The error bars of the Cohen's *D*s represent their respective 95% confidence intervals

Next, we applied the young‐train and old‐train behavioral prediction models to the test data sets of all three age groups and calculated the RMSE ratios between age groups. Figure 5a,b illustrates these RMSE ratios. A paired‐samples *t*‐test, comparing the RMSE ratios using the young and old as the baseline, collapsing across modality and generalized age‐groups, revealed a statistically significant and moderate effect‐size for the difference (*t*[87] = 4.09, *p* < .001, *d* = .48) in the RMSE ratios between the young‐baseline and old‐baseline scenarios. This suggests that, overall, BBAs derived from the old were significantly more age‐generalizable compared to that of the young. Furthermore, one would also notice an obvious disparity between the young‐to‐middle and young‐to‐old RMSE ratios (*t*[43] = 10.15, *p* < .001, *d* = 1.82), whereas the disparity between the old‐to‐young and old‐to‐middle RMSE ratios was much smaller, though still statistically significant (*t*[43] = 3.50, *p* = .001, *d* = .37). These findings converged to suggest that the young‐derived, relative to the old‐derived, brain‐behavior associations were highly age specific.

### 3.3 Does age‐generalizability depend on the age‐related differences in behavioral scores?

Previous research has suggested the age‐generalizability of brain‐behavior associations was partly influenced by the between‐age‐group differences in behavioral scores (Yu & Fischer, 2022); that is if the behavioral scores differed tremendously between the young and old, one would expect the young‐to‐old and old‐to‐young generalizability to be low. Indeed, our results in Table 2 showed that the RMSE ratios correlated highly with the between‐age‐group differences in behavioral scores as measured using Cohen's *D* for most scenarios, except in that of young vs middle age‐groups (see Figure 5c,d).

**TABLE 2:** Correlations between RMSE ratios and Cohen's *D*

Modality	Pearson's correlation between			
	Ratio_young‐to‐old_	Ratio_young‐to‐mid_	Ratio_old‐to‐young_	Ratio_old‐to‐mid_
	*D*_young vs old_	*D*_young vs mid_	*D*_old vs young_	*D*_old vs mid_
SC	−0.84	−0.16	−0.83	−0.51
rsFC	−0.80	−0.16	−0.83	−0.52
GM	−0.85	−0.12	−0.61	−0.43
Stacked	−0.76	−0.10	−0.73	−0.34

*Note*: *D*, absolute value of Cohen's *D*; Ratio, root mean square ratio.

Additionally, we would like to highlight the interesting fact that the young‐to‐old and old‐to‐young RMSE ratios differed significantly (*t*[43] = 7.09, *p* < .001, *d* = 1.30) despite the differences in behavioral scores being a constant in both generalization scenarios. This meant that the asymmetric pattern of age‐generalizability (i.e., young generalized poorly to old, but old generalized well to young), was very likely to be entirely independent of the between‐age‐group differences in behavioral scores.

### 3.4 Effect of modality

Finally, we collapsed the prediction metrics across age groups to examine if the modality (i.e., GM, rsFC, SC, and stacked) of the brain‐behavior association may have a significant influence on the RMSE ratio values. Repeated measures ANOVAs, with the behavioral outcomes and the modalities as the within and between factors respectively, did not reveal significant differences between modalities in the RMSE ratios (*p*s > .89; partial *η* ^2^ ≤ .007).

## 4 DISCUSSION

In the current study, we examined how the young and old derived BBAs would generalize to other age‐groups. Specifically, we hypothesized that the greater the age differences between the train and test samples, the poorer the train‐derived BBA would generalize to the test sample. Our results partially supported this hypothesis. For instance, when the young were used as the training sample, the results revealed a graded pattern of model generalization across age‐groups. As expected, across most behavioral outcomes, young‐to‐young generalizability was the highest, followed by young‐to‐middle, and then young‐to‐old. On the other hand, when the old was used as the training sample, a few BBAs (i.e., UnfamFace_Recog and VSTM) demonstrated similar patterns of graded generalizability across age‐groups, albeit to a much smaller extent. However, for most of the other BBAs, the disparity between the old‐to‐young, old‐to‐middle, and old‐to‐old generalizability was neglible. Overall, these results suggest that young‐derived BBAs were highly age‐specific while the old‐derived BBAs were likely to be generalizable across other age‐groups.

The asymmetric pattern of age‐generalizability observed for most BBAs is perhaps the most intriguing result in this study. To account for this, we propose that individual differences in cognitive abilities across the lifespan can be explained by a complementary combination of age‐general and age‐specific neurobiological processes. The former occurs throughout the lifespan and is theorized to consistently affect brain and behavior, giving rise to consistent BBAs across age‐groups. The latter affects specific age‐groups, producing BBAs that are only observed in their particular age‐group. Hence, different age‐groups can be predicted fairly well by prediction models that exploit the age‐general processes, however, prediction models that are heavily driven by age‐specific processes are unable to generalize beyond the age‐group they are derived from. Essentially, the asymmetric generalization results meant that the BBAs derived from the young are predominantly influenced by age‐specific processes, whereas those derived from the old are largely influenced by age‐general processes.

One of such age‐general processes relates to experience‐dependent neuroplasticity. Cognitive abilities can be enhanced with repeated practice. Such improvements would correspond to plastic changes in the brain, both in terms of structure and function (Park & Bischof, 2013). On the other hand, the degeneration of brain networks or atrophy of certain brain structures, which could occur in the context of aging or health‐related factors (e.g., stress), would correspondingly lead to a decline in cognitive functions (Oh et al., 2014). Both neuroplasticity and neurodegeneration occur across the lifespan and would give rise to individual differences in brain and behavior, such that a “positive” BBA can be observed. That is, cognitive abilities are positively associated with GM volume/CT in various brain regions and perhaps to some extent, the functional and structural connectivity between brain regions as well.

While neuroplasticity and neurodegeneration implicate individual differences in brain and behavior in all three studied age‐groups, the young age‐group may be additionally confounded by the relatively age‐specific neurodevelopmental trend, which would give rise to “negative” BBAs. For instance, Schlichting et al. (2017) have described a nonlinear hippocampal developmental trend from early childhood to adulthood, with the hippocampus' head volume increasing during childhood, before it decreases during early adulthood. Moreover, smaller hippocampal volumes in early adulthood were associated with better memory abilities, which the authors interpreted to be related to the pruning of extraneous connections leading to increased hippocampal processing efficiency. Although the exact pathways underlying this remodeling process are still not completely characterized, this is largely consistent with our results, since within the young age‐group, smaller subcortical volumes were also associated with better scores on a memory‐related test as shown in Figure 4.

Age‐specific neurobiological processes do implicate BBAs in the old‐age groups as well. The maintenance of cognitive abilities during advanced age have been attributed to age‐related reorganization and dedifferentiation of brain networks, which can be considered as age‐specific processes that disproportionately implicate the elderly more so than the young, and can possibly account for the loss in the generalizability of old‐derived BBAs in the younger age‐groups. However, this loss did not seem to be as significant as that observed when the young‐derived BBAs were generalized to the older age‐groups, suggesting these old‐age specific processes had minimal influence on the BBAs. In relation to this, it can also be argued that the reorganization and dedifferentiation of brain networks are not that age‐specific after all. For instance, CAM‐CAN consortium et al. (2017) demonstrated that cognitively high performing older adults tend to possess more youth‐like functional characteristics, which is likely to optimize brain function to support a healthy aging process and maintain, as far as possible, one's cognitive functions at a level similar to those observed in the young. Furthermore, the reorganization of the brain's networks that occur during healthy aging could be considered as an adaptive mechanism accounting for its resilience and can be used not only to understand how someone copes with stress (in its broadest definition) throughout life, but also to illustrate how neuroplastic changes to the brain structural and functional networks take place consistently across the lifespan (Gonzalez‐Escamilla et al., 2018).

We observed there were a few interesting instances where models of BBAs derived from the young or old, predicted scores more accurately in other age‐groups than the age‐group they were derived from. For instance, the FamFace_Occupation model derived from the young, predicted scores more accurately (RMSE ratio > 1) in the middle age‐group, than in the young age‐group. This could mean that behavioral measurement error in the derived sample was much higher than in the generalized sample. This measurement error can come from many sources, such as the greater ceiling effect observed in the young, the confounding influences of better education levels, and media exposure (Kramer et al., 2018) among the young. One could make a similar observation with the RT_choice model derived from the old; this old‐derived model predicted scores more accurately in the young and middle age‐groups than in the old. In this reaction time task, participants had to press the correct button as indicated by the corresponding black circles; the measurement errors are likely to be explained by the fine‐motor/motor‐related impairments prevalent in the old (Smith et al., 1999). Additionally, the random train‐test partitioning in young and old data sets was likely to produce test folds that were dissimilar to the training folds in terms of the range of age and behavioral scores—this too is likely to add to the prediction error in the test folds of the same age‐group.

On the whole, it appears that our prediction and generalizability‐related results were not significantly different across modalities; this is largely consistent with our previous work (Yu & Fischer, 2022). Taken together, these results possibly suggest that different modalities of input features were highly correlated with each other, and hence did not significantly outperform each other in predicting behavioral outcomes. Nevertheless, given the relatively narrow selection of behavioral outcomes in this study, we do not want to rule out the possibility that some neuroimaging modalities may be more predictive of certain behavioral outcomes than others. Given that rsFC is highly dynamic in nature (Hutchison et al., 2013), changes to rsFC will take place on a much shorter time scale than those of structural features. To this end, it is possible that state‐like behavioral outcomes may be better predicted by rsFC features, whereas trait‐like behavioral outcomes are likely to be better predicted by structural features. In the current study, we could not test such a hypothesis since the behavioral outcomes did not vary meaningfully on the state–trait dimension; this remains to be examined in future research.

In the construction of the structural and functional connectomes, we did not to use the same atlas for both; these different atlases thus made it difficult for one to compare the connectivity patterns between both types of connectomes. Instead, we used atlases that were optimized for their respective modalities. While functional connectomes are best constructed using atlases of finer granularity in order to optimize behavioral predictions (Wu et al., 2021), structural connectomes are more appropriately constructed using atlases of coarser granularity such as the AAL‐90 atlas. If we had used the same brainnetome atlas (used to constructing the functional connectome) to construct the structural connectome, the resulting SC matrices will be extremely sparse (i.e., too many zero values) for it to be analyzed meaningfully. Although the choice of atlas can influence accuracy of behavioral predictions, in the current study we were more interested in ratio of the prediction metrics obtained from different age‐groups, rather than the absolute prediction metrics in a specific age‐group. In this regard, we would expect the use of different atlases to improve or worsen predictions in different age‐groups to a similar extent, thus keeping this ratio relatively constant, as demonstrated in a previous study (Yu & Fischer, 2022).

The current study is subjected to some limitations. First, we had to exclude a large number of participants (*N* = 101) due to excessive head motion during their rsfMRI scans. Furthermore, the excluded sample (age_mean_ = 65.1, SD = 15.7) were significantly older (*t*[160] = 7.08, *p* < .001, *d* = .68) than the included sample (age_mean_ = 52.9, SD = 18.5), suggesting a possible selection bias in the sample inclusion. In relation to this, increased head motion during rsfMRI scans is typically observed among the older age‐groups (Saccà et al., 2021). Next, we were not able to include many other behavioral scores in the CAM‐CAN data set due to incomplete and missing data. Additionally, some of these behavioral scores correlated highly with each other (see supplementary materials), especially among the different scores derived from the same behavioral tasks. This suggests that we did not include a range of behavioral measures as diverse as we desired. Finally, due to the manner in which we partitioned the CAM‐CAN data set, the age‐range in the three age‐groups can be fairly large (>20 years), and these individual differences in age can potentially confound BBAs.

Overall, our findings present some major implications for research involving brain‐based behavioral predictions. We showed that behavioral prediction models derived from young participants' multimodal neuroimaging data performed poorly on older age‐groups as the age gaps increased. The young age‐group (i.e., college students) is perhaps the easiest to recruit relative to the other age‐groups. Furthermore, publicly accessible neuroimaging data sets, such that those hosted on OpenNeuro are collected mostly from participants aged ≤30 (Markiewicz et al., 2021). Most neuroimaging research has been and will continue to be carried out on young samples. In light of our findings, we suggest that future researchers who utilize study samples consisting of mostly young subjects, explicitly recognize the age‐generalizability limits of their brain‐behavior findings. We also caution against using evidence from young age‐group studies to support scientific arguments and theories involving brain‐behavior relationships in the samples constituted by elderly individuals.

## CONFLICT OF INTEREST

The authors declare no competing financial interests.

## Supporting information


**Appendix S1** Supporting Information

## Data Availability

The preprocessed data analyzed in this study are available at https://osf.io/wbhkg/. The raw behavioral and neuroimaging data used in this study can be obtained from the CamCAN repository via a data access application (https://camcan-archive.mrc-cbu.cam.ac.uk/dataaccess/).

## References

[hbm26035-bib-0001] Battaglini, M. , Gentile, G. , Luchetti, L. , Giorgio, A. , Vrenken, H. , Barkhof, F. , Cover, K. S. , Bakshi, R. , Chu, R. , Sormani, M. P. , Enzinger, C. , Ropele, S. , Ciccarelli, O. , Wheeler‐Kingshott, C. , Yiannakas, M. , Filippi, M. , Rocca, M. A. , Preziosa, P. , Gallo, A. , … De Stefano, N. (2019). Lifespan normative data on rates of brain volume changes. Neurobiology of Aging, 81, 30–37. 10.1016/j.neurobiolaging.2019.05.010 31207467

[hbm26035-bib-0002] CAM‐CAN consortium , Samu, D. , Campbell, K. L. , Tsvetanov, K. A. , Shafto, M. A. , & Tyler, L. K. (2017). Preserved cognitive functions with age are determined by domain‐dependent shifts in network responsivity. Nature Communications, 8(1), 14743. 10.1038/ncomms14743 PMC5424147 28480894

[hbm26035-bib-0003] Chan, M. Y. , Park, D. C. , Savalia, N. K. , Petersen, S. E. , & Wig, G. S. (2014). Decreased segregation of brain systems across the healthy adult lifespan. Proceedings of the National Academy of Sciences of the United States of America, 111(46), E4997–E5006. 25368199 10.1073/pnas.1415122111 PMC4246293

[hbm26035-bib-0004] Cox, R. W. (1996). AFNI: Software for analysis and visualization of functional magnetic resonance neuroimages. Computers and Biomedical Research, 29(3), 162–173. 8812068 10.1006/cbmr.1996.0014

[hbm26035-bib-0005] Dale, A. , Fischl, B. , & Sereno, M. I. (1999). Cortical surface‐based analysis: I. Segmentation and surface reconstruction. NeuroImage, 9(2), 179–194. 10.1006/nimg.1998.0395 9931268

[hbm26035-bib-0006] Destrieux, C. , Fischl, B. , Dale, A. , & Halgren, E. (2010). Automatic parcellation of human cortical gyri and sulci using standard anatomical nomenclature. NeuroImage, 53(1), 1–15. 20547229 10.1016/j.neuroimage.2010.06.010 PMC2937159

[hbm26035-bib-0007] Dhollander, T. , & Connelly, A. (2016). A novel iterative approach to reap the benefits of multi‐tissue CSD from just single‐shell (+b = 0) diffusion MRI data. 24th Int. Soc. Magn. Reson. Med, 24, 3010.

[hbm26035-bib-0008] Esteban, O. , Markiewicz, C. J. , Blair, R. W. , Moodie, C. A. , Isik, A. I. , Erramuzpe, A. , Kent, J. D. , Goncalves, M. , DuPre, E. , & Snyder, M. (2019). fMRIPrep: A robust preprocessing pipeline for functional MRI. Nature Methods, 16(1), 111–116. 30532080 10.1038/s41592-018-0235-4 PMC6319393

[hbm26035-bib-0009] Fan, L. , Li, H. , Zhuo, J. , Zhang, Y. , Wang, J. , Chen, L. , Yang, Z. , Chu, C. , Xie, S. , & Laird, A. R. (2016). The human brainnetome atlas: A new brain atlas based on connectional architecture. Cerebral Cortex, 26(8), 3508–3526. 27230218 10.1093/cercor/bhw157 PMC4961028

[hbm26035-bib-0010] Fischl, B. , & Dale, A. M. (2000). Measuring the thickness of the human cerebral cortex from magnetic resonance images. Proceedings of the National Academy of Sciences of the United States of America, 97(20), 11050–11055. 10984517 10.1073/pnas.200033797 PMC27146

[hbm26035-bib-0011] Fischl, B. , Liu, A. , & Dale, A. M. (2001). Automated manifold surgery: Constructing geometrically accurate and topologically correct models of the human cerebral cortex. IEEE Medical Imaging, 20(1), 70–80. 11293693 10.1109/42.906426

[hbm26035-bib-0012] Fischl, B. , Salat, D. H. , Busa, E. , Albert, M. , Dieterich, M. , Haselgrove, C. , van der Kouwe, A. , Killiany, R. , Kennedy, D. , Klaveness, S. , Montillo, A. , Makris, N. , Rosen, B. , & Dale, A. M. (2002). Whole brain segmentation: Automated labeling of neuroanatomical structures in the human brain. Neuron, 33, 341–355. 11832223 10.1016/s0896-6273(02)00569-x

[hbm26035-bib-0013] Fischl, B. , Salat, D. H. , van der Kouwe, A. J. W. , Makris, N. , Ségonne, F. , Quinn, B. T. , & Dale, A. M. (2004). Sequence‐independent segmentation of magnetic resonance images. NeuroImage, 23(Suppl 1), S69–S84. 10.1016/j.neuroimage.2004.07.016 15501102

[hbm26035-bib-0014] Fischl, B. , Sereno, M. I. , & Dale, A. (1999). Cortical surface‐based analysis: II: Inflation, flattening, and a surface‐based coordinate system. NeuroImage, 9(2), 195–207. 10.1006/nimg.1998.0396 9931269

[hbm26035-bib-0015] Gao, M. , Wong, C. H. Y. , Huang, H. , Shao, R. , Huang, R. , Chan, C. C. H. , & Lee, T. M. C. (2020). Connectome‐based models can predict processing speed in older adults. NeuroImage, 223, 117290. 10.1016/j.neuroimage.2020.117290 32871259

[hbm26035-bib-0016] Gao, S. , Greene, A. S. , Constable, R. T. , & Scheinost, D. (2019). Combining multiple connectomes improves predictive modeling of phenotypic measures. NeuroImage, 201, 116038. 10.1016/j.neuroimage.2019.116038 31336188 PMC6765422

[hbm26035-bib-0017] Gonzalez‐Escamilla, G. , Muthuraman, M. , Chirumamilla, V. C. , Vogt, J. , & Groppa, S. (2018). Brain networks reorganization during maturation and healthy aging‐emphases for resilience. Frontiers in Psychiatry, 9, 601. 10.3389/fpsyt.2018.00601 30519196 PMC6258799

[hbm26035-bib-0018] Greve, D. N. , & Fischl, B. (2009). Accurate and robust brain image alignment using boundary‐based registration. NeuroImage, 48(1), 63–72. 19573611 10.1016/j.neuroimage.2009.06.060 PMC2733527

[hbm26035-bib-0019] Hutchison, R. M. , Womelsdorf, T. , Allen, E. A. , Bandettini, P. A. , Calhoun, V. D. , Corbetta, M. , Della Penna, S. , Duyn, J. H. , Glover, G. H. , Gonzalez‐Castillo, J. , Handwerker, D. A. , Keilholz, S. , Kiviniemi, V. , Leopold, D. A. , de Pasquale, F. , Sporns, O. , Walter, M. , & Chang, C. (2013). Dynamic functional connectivity: Promise, issues, and interpretations. NeuroImage, 80, 360–378. 10.1016/j.neuroimage.2013.05.079 23707587 PMC3807588

[hbm26035-bib-0020] Jenkinson, M. , Bannister, P. , Brady, M. , & Smith, S. (2002). Improved optimization for the robust and accurate linear registration and motion correction of brain images. NeuroImage, 17(2), 825–841. 12377157 10.1016/s1053-8119(02)91132-8

[hbm26035-bib-0021] Kellner, E. , Dhital, B. , Kiselev, V. G. , & Reisert, M. (2016). Gibbs‐ringing artifact removal based on local subvoxel‐shifts. Magnetic Resonance in Medicine, 76(5), 1574–1581. 10.1002/mrm.26054 26745823

[hbm26035-bib-0022] Knyazev, G. G. , Volf, N. V. , & Belousova, L. V. (2015). Age‐related differences in electroencephalogram connectivity and network topology. Neurobiology of Aging, 36(5), 1849–1859. 10.1016/j.neurobiolaging.2015.02.007 25766772

[hbm26035-bib-0023] Koen, J. D. , Srokova, S. , & Rugg, M. D. (2020). Age‐related neural dedifferentiation and cognition. Current Opinion in Behavioral Sciences, 32, 7–14. 10.1016/j.cobeha.2020.01.006 32095492 PMC7039299

[hbm26035-bib-0024] Kramer, R. S. S. , Young, A. W. , & Burton, A. M. (2018). Understanding face familiarity. Cognition, 172, 46–58. 10.1016/j.cognition.2017.12.005 29232594

[hbm26035-bib-0025] Markiewicz, C. J. , Gorgolewski, K. J. , Feingold, F. , Blair, R. , Halchenko, Y. O. , Miller, E. , Hardcastle, N. , Wexler, J. , Esteban, O. , Goncavles, M. , Jwa, A. , & Poldrack, R. (2021). The OpenNeuro resource for sharing of neuroscience data. eLife, 10, e71774. 10.7554/eLife.71774 34658334 PMC8550750

[hbm26035-bib-0026] Oh, H. , Madison, C. , Villeneuve, S. , Markley, C. , & Jagust, W. J. (2014). Association of gray matter atrophy with age, β‐amyloid, and cognition in aging. Cerebral Cortex, 24(6), 1609–1618. 10.1093/cercor/bht017 23389995 PMC4014182

[hbm26035-bib-0027] Park, D. C. , & Bischof, G. N. (2013). The aging mind: Neuroplasticity in response to cognitive training. Dialogues in Clinical Neuroscience, 15(1), 109–119. 23576894 10.31887/DCNS.2013.15.1/dpark PMC3622463

[hbm26035-bib-0046] Power, J. D., Mitra, A., Laumann, T. O., Snyder, A. Z., Schlaggar, B. L., & Petersen, S. E. (2014). Methods to detect, characterize, and remove motion artifact in resting state fMRI. *Neuroimage*, 84, 320–341. 10.1016/j.neuroimage.2013.08.048 PMC3849338 23994314

[hbm26035-bib-0047] Rolls, E. T., Joliot, M., & Tzourio‐Mazoyer, N. (2015). Implementation of a new parcellation of the orbitofrontal cortex in the automated anatomical labeling atlas. *Neuroimage*, 122, 1–5. 10.1016/j.neuroimage.2015.07.075 26241684

[hbm26035-bib-0028] Rubinov, M. , & Sporns, O. (2010). Complex network measures of brain connectivity: Uses and interpretations. NeuroImage, 52(3), 1059–1069. 10.1016/j.neuroimage.2009.10.003 19819337

[hbm26035-bib-0029] Saccà, V. , Sarica, A. , Quattrone, A. , Rocca, F. , Quattrone, A. , & Novellino, F. (2021). Aging effect on head motion: A machine learning study on resting state fMRI data. Journal of Neuroscience Methods, 352, 109084. 10.1016/j.jneumeth.2021.109084 33508406

[hbm26035-bib-0030] Salat, D. H. , Tuch, D. S. , Greve, D. N. , Van Der Kouwe, A. J. W. , Hevelone, N. D. , Zaleta, A. K. , Rosen, B. R. , Fischl, B. , Corkin, S. , & Rosas, H. D. (2005). Age‐related alterations in white matter microstructure measured by diffusion tensor imaging. Neurobiology of Aging, 26(8), 1215–1227. 15917106 10.1016/j.neurobiolaging.2004.09.017

[hbm26035-bib-0031] Schlichting, M. L. , Guarino, K. F. , Schapiro, A. C. , Turk‐Browne, N. B. , & Preston, A. R. (2017). Hippocampal structure predicts statistical learning and associative inference abilities during development. Journal of Cognitive Neuroscience, 29(1), 37–51. 27575916 10.1162/jocn_a_01028 PMC5130612

[hbm26035-bib-0032] Segonne, F. , Dale, A. M. , Busa, E. , Glessner, M. , Salat, D. , Hahn, H. K. , & Fischl, B. (2004). A hybrid approach to the skull stripping problem in MRI. NeuroImage, 22(3), 1060–1075. 10.1016/j.neuroimage.2004.03.032 15219578

[hbm26035-bib-0033] Segonne, F. , Pacheco, J. , & Fischl, B. (2007). Geometrically accurate topology‐correction of cortical surfaces using nonseparating loops. IEEE Transactions on Medical Imaging, 26, 518–529. 17427739 10.1109/TMI.2006.887364

[hbm26035-bib-0034] Shafto, M. A. , Tyler, L. K. , Dixon, M. , Taylor, J. R. , Rowe, J. B. , Cusack, R. , Calder, A. J. , Marslen‐Wilson, W. D. , Duncan, J. , Dalgleish, T. , Henson, R. N. , Brayne, C. , Matthews, F. E. , & CAM‐CAN . (2014). The Cambridge Centre for Ageing and Neuroscience (Cam‐CAN) study protocol: A cross‐sectional, lifespan, multidisciplinary examination of healthy cognitive ageing. BMC Neurology, 14, 204. 10.1186/s12883-014-0204-1 25412575 PMC4219118

[hbm26035-bib-0035] Sled, J. G. , Zijdenbos, A. P. , & Evans, A. C. (1998). A nonparametric method for automatic correction of intensity nonuniformity in MRI data. IEEE Transactions on Medical Imaging, 17, 87–97. 9617910 10.1109/42.668698

[hbm26035-bib-0036] Smith, C. D. , Umberger, G. H. , Manning, E. L. , Slevin, J. T. , Wekstein, D. R. , Schmitt, F. A. , Markesbery, W. R. , Zhang, Z. , Gerhardt, G. A. , Kryscio, R. J. , & Gash, D. M. (1999). Critical decline in fine motor hand movements in human aging. Neurology, 53(7), 1458. 10.1212/WNL.53.7.1458 10534251

[hbm26035-bib-0037] Smith, R. E. , Tournier, J.‐D. , Calamante, F. , & Connelly, A. (2012). Anatomically‐constrained tractography: Improved diffusion MRI streamlines tractography through effective use of anatomical information. NeuroImage, 62(3), 1924–1938. 10.1016/j.neuroimage.2012.06.005 22705374

[hbm26035-bib-0038] Stumme, J. , Jockwitz, C. , Hoffstaedter, F. , Amunts, K. , & Caspers, S. (2020). Functional network reorganization in older adults: Graph‐theoretical analyses of age, cognition and sex. NeuroImage, 214, 116756. 10.1016/j.neuroimage.2020.116756 32201326

[hbm26035-bib-0039] Taylor, J. R. , Williams, N. , Cusack, R. , Auer, T. , Shafto, M. A. , Dixon, M. , Tyler, L. K. , CAM‐CAN , & Henson, R. N. (2017). The Cambridge Centre for Ageing and Neuroscience (Cam‐CAN) data repository: Structural and functional MRI, MEG, and cognitive data from a cross‐sectional adult lifespan sample. NeuroImage, 144, 262–269. 10.1016/j.neuroimage.2015.09.018 26375206 PMC5182075

[hbm26035-bib-0040] Tournier, J. D. , Smith, R. , Raffelt, D. , Tabbara, R. , Dhollander, T. , Pietsch, M. , Christiaens, D. , Jeurissen, B. , Yeh, C. H. , & Connelly, A. (2019). MRtrix3: A fast, flexible and open software framework for medical image processing and visualisation. NeuroImage, 202, 116137. 10.1016/j.neuroimage.2019.116137 31473352

[hbm26035-bib-0041] Tustison, N. J. , Avants, B. B. , Cook, P. A. , Zheng, Y. , Egan, A. , Yushkevich, P. A. , & Gee, J. C. (2010). N4ITK: Improved N3 bias correction. IEEE Transactions on Medical Imaging, 29(6), 1310–1320. 10.1109/TMI.2010.2046908 20378467 PMC3071855

[hbm26035-bib-0042] Tzourio‐Mazoyer, N. , Landeau, B. , Papathanassiou, D. , Crivello, F. , Etard, O. , Delcroix, N. , Mazoyer, B. , & Joliot, M. (2002). Automated anatomical labeling of activations in SPM using a macroscopic anatomical parcellation of the MNI MRI single‐subject brain. NeuroImage, 15(1), 273–289. 10.1006/nimg.2001.0978 11771995

[hbm26035-bib-0043] Veraart, J. , Novikov, D. S. , Christiaens, D. , Ades‐aron, B. , Sijbers, J. , & Fieremans, E. (2016). Denoising of diffusion MRI using random matrix theory. NeuroImage, 142, 394–406. 10.1016/j.neuroimage.2016.08.016 27523449 PMC5159209

[hbm26035-bib-0044] Wu, J. , Eickhoff, S. B. , Hoffstaedter, F. , Patil, K. R. , Schwender, H. , Yeo, B. T. T. , & Genon, S. (2021). A connectivity‐based psychometric prediction framework for brain–behavior relationship studies. Cerebral Cortex, 31(8), 3732–3751. 10.1093/cercor/bhab044 33884421 PMC8660224

[hbm26035-bib-0045] Yu, J. , & Fischer, N. L. (2022). Age‐specificity and generalization of behavior‐associated structural and functional networks and their relevance to behavioral domains. Human Brain Mapping, 43(8), 1–14. 10.1002/hbm.25759 PMC9057094 35274793

