# Supplementary material for: Asymmetric generalizability of multimodal brain‐behavior associations across age‐groups
Source: Hum Brain Mapp. 2022 Jul 30;43(18):5593–604. doi: 10.1002/hbm.26035 (PMC9704787; doi:10.1002/hbm.26035)
Supplement: Supplementary file 1 — Appendix S1 Supporting Information [file HBM-43-5593-s001.docx]

**Correlation heatmap of all studied variables**


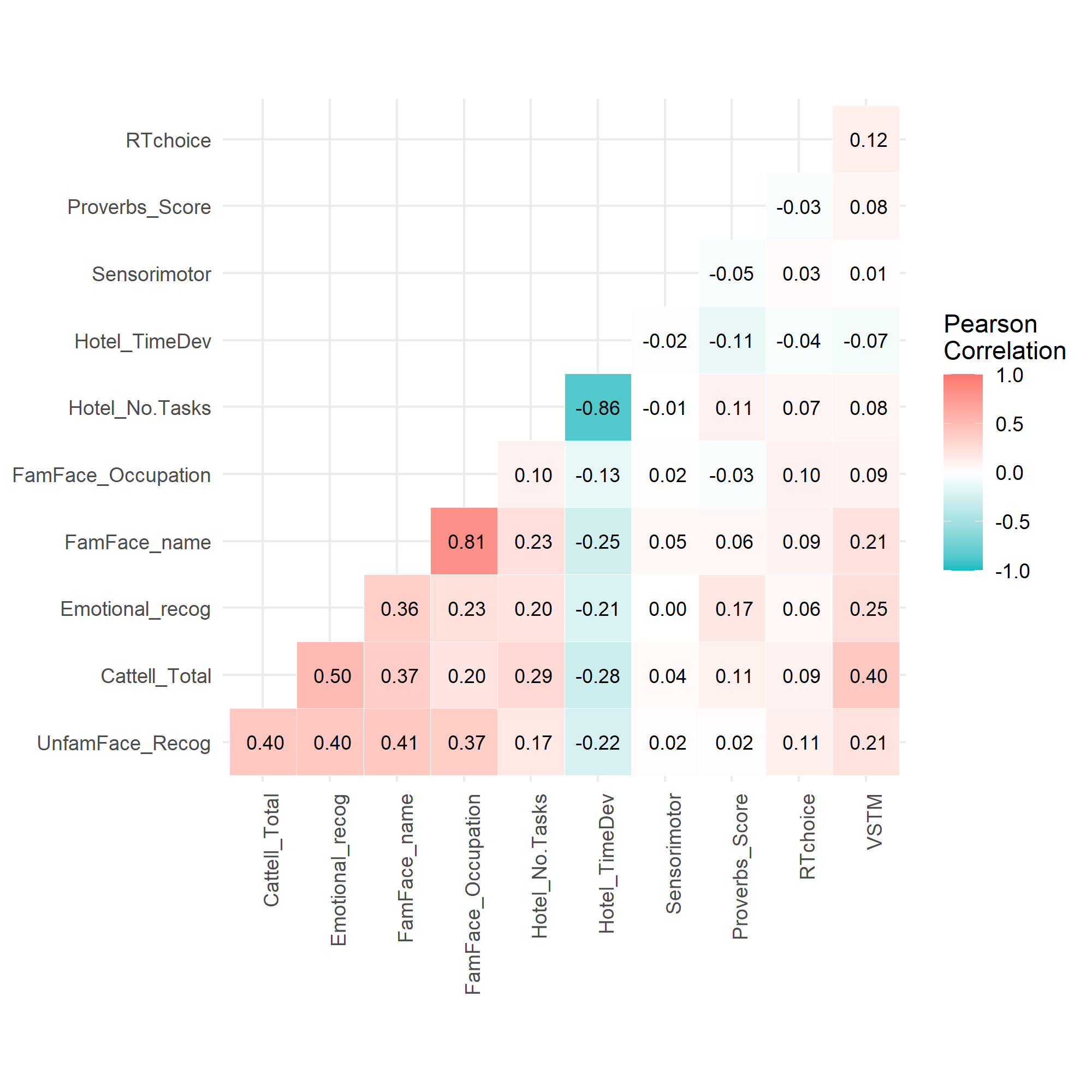


**Beta coefficients plots**

**Cattell_Total**


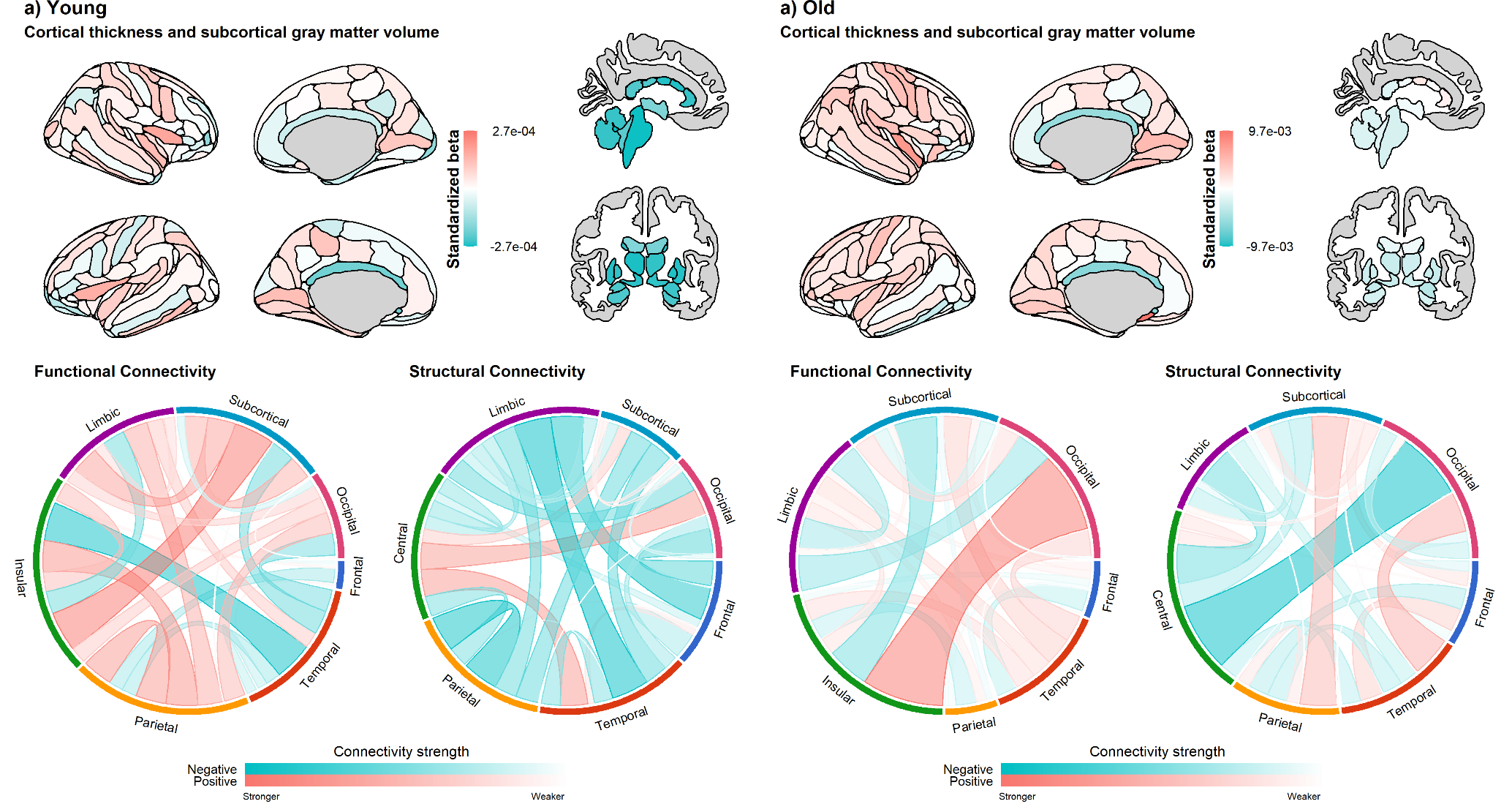


**Emotional_recog**


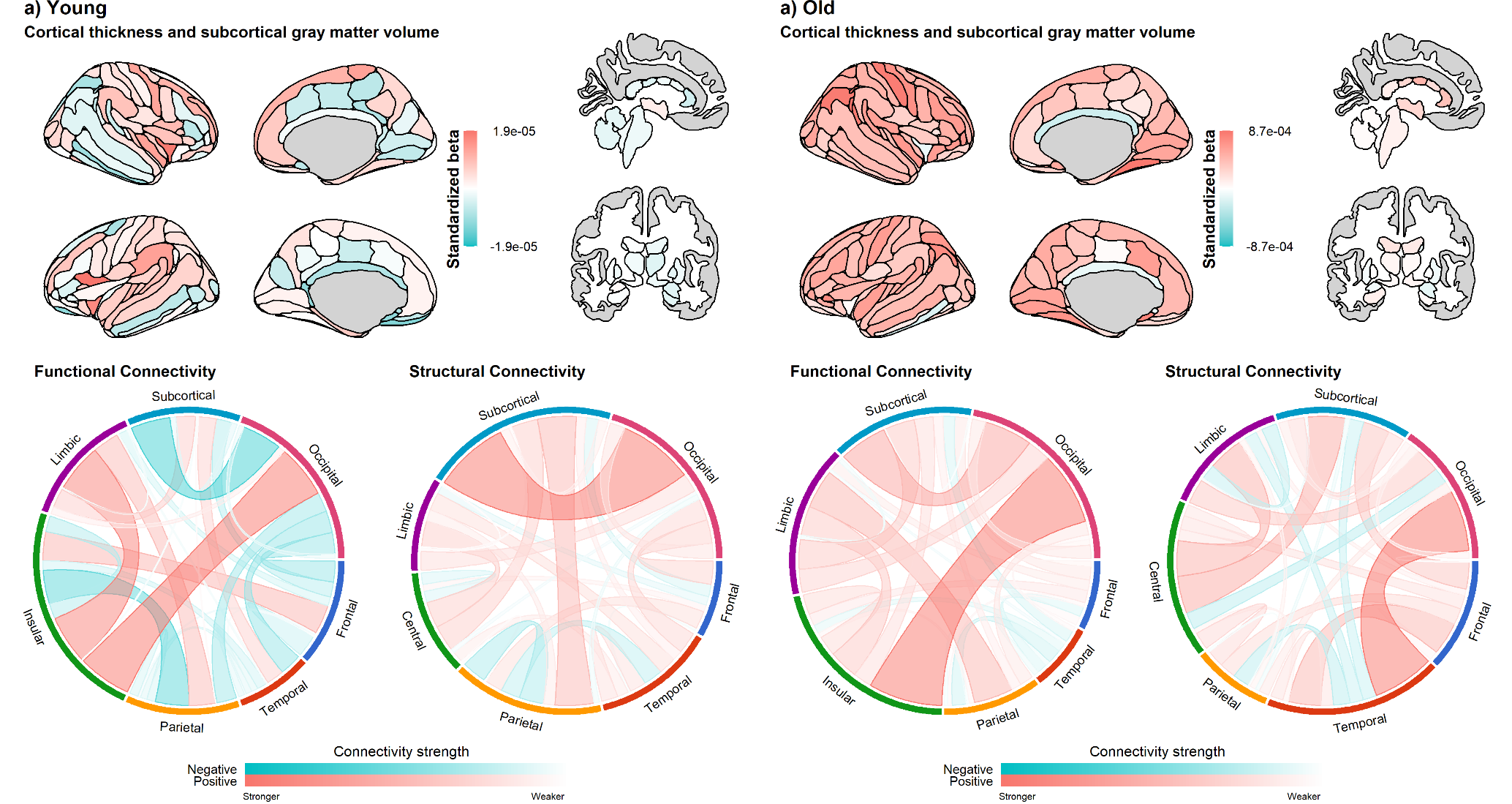


**FamFace_name**

**
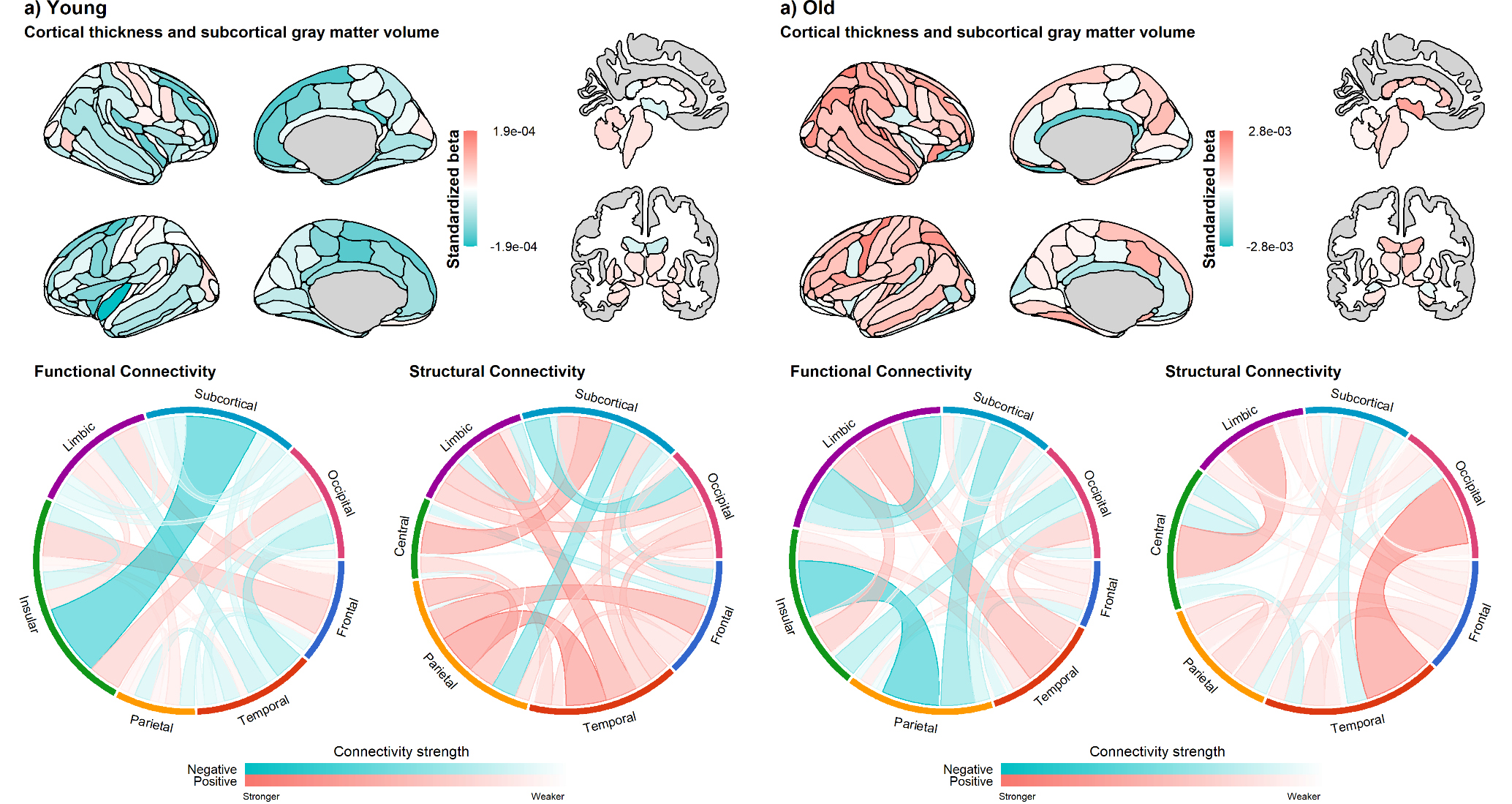
**

**FamFace_Occupation**

**
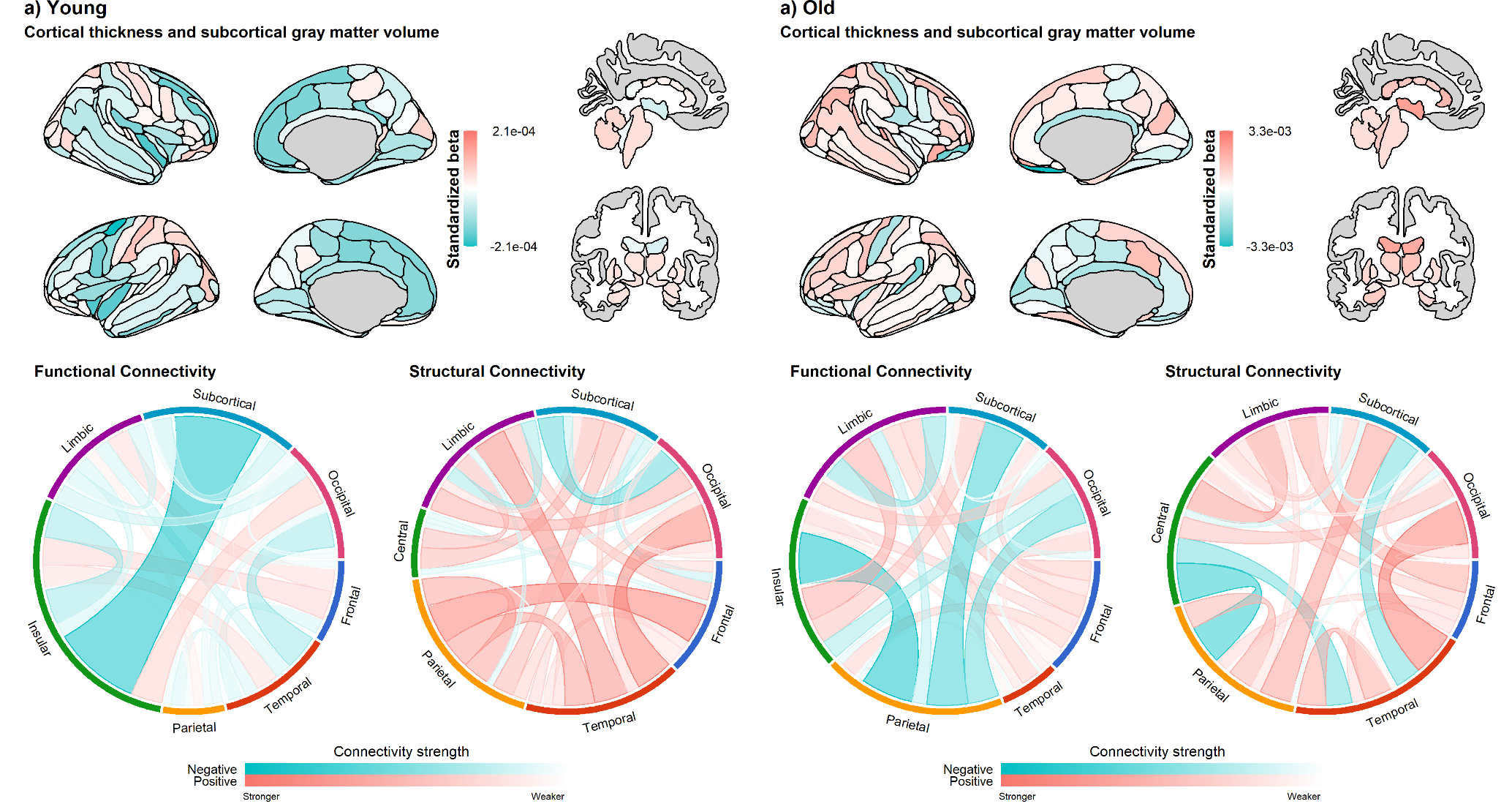
**

**Hotel_No.Tasks**

**
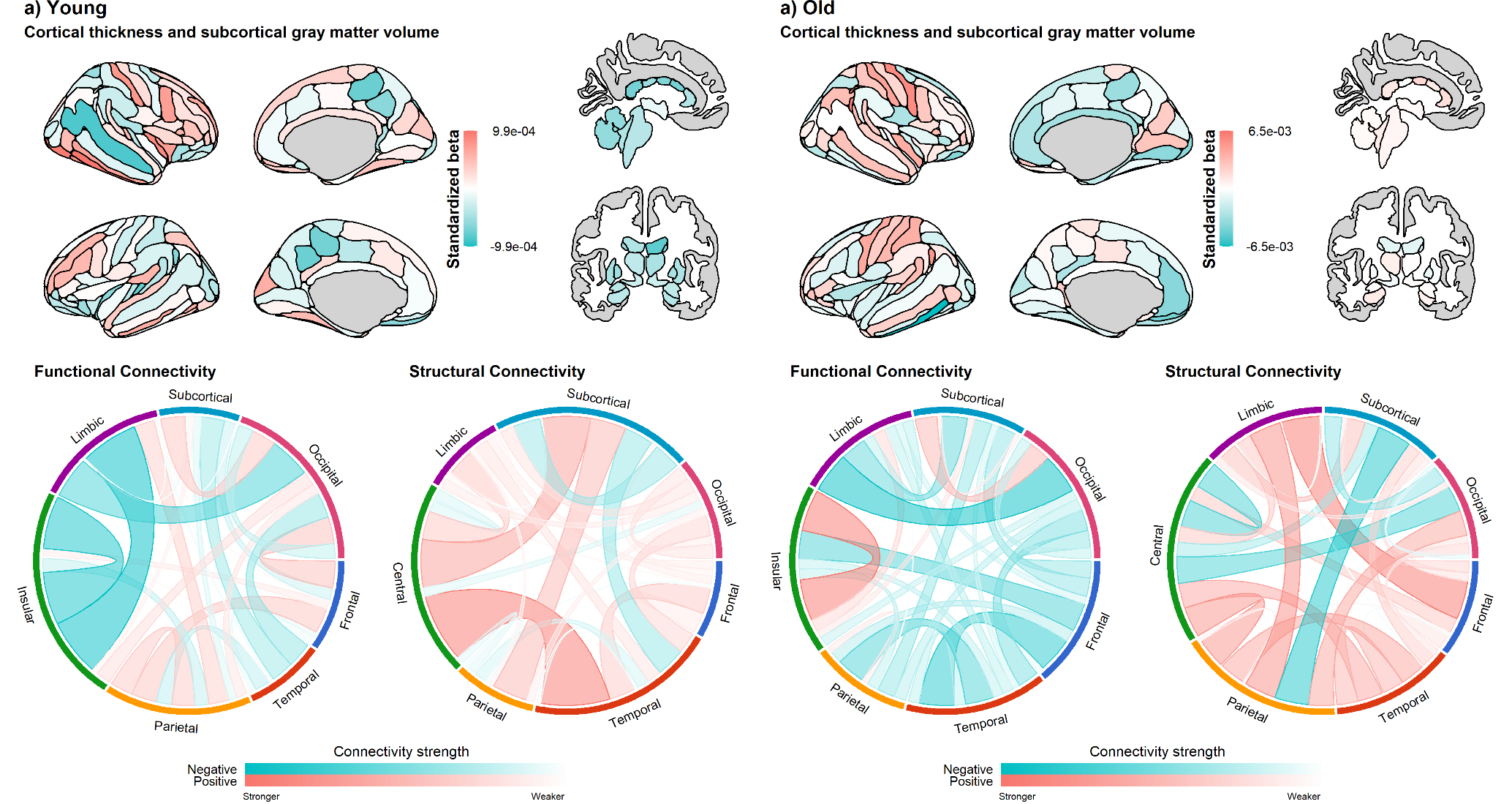
**

**Hotel_TimeDev**

**
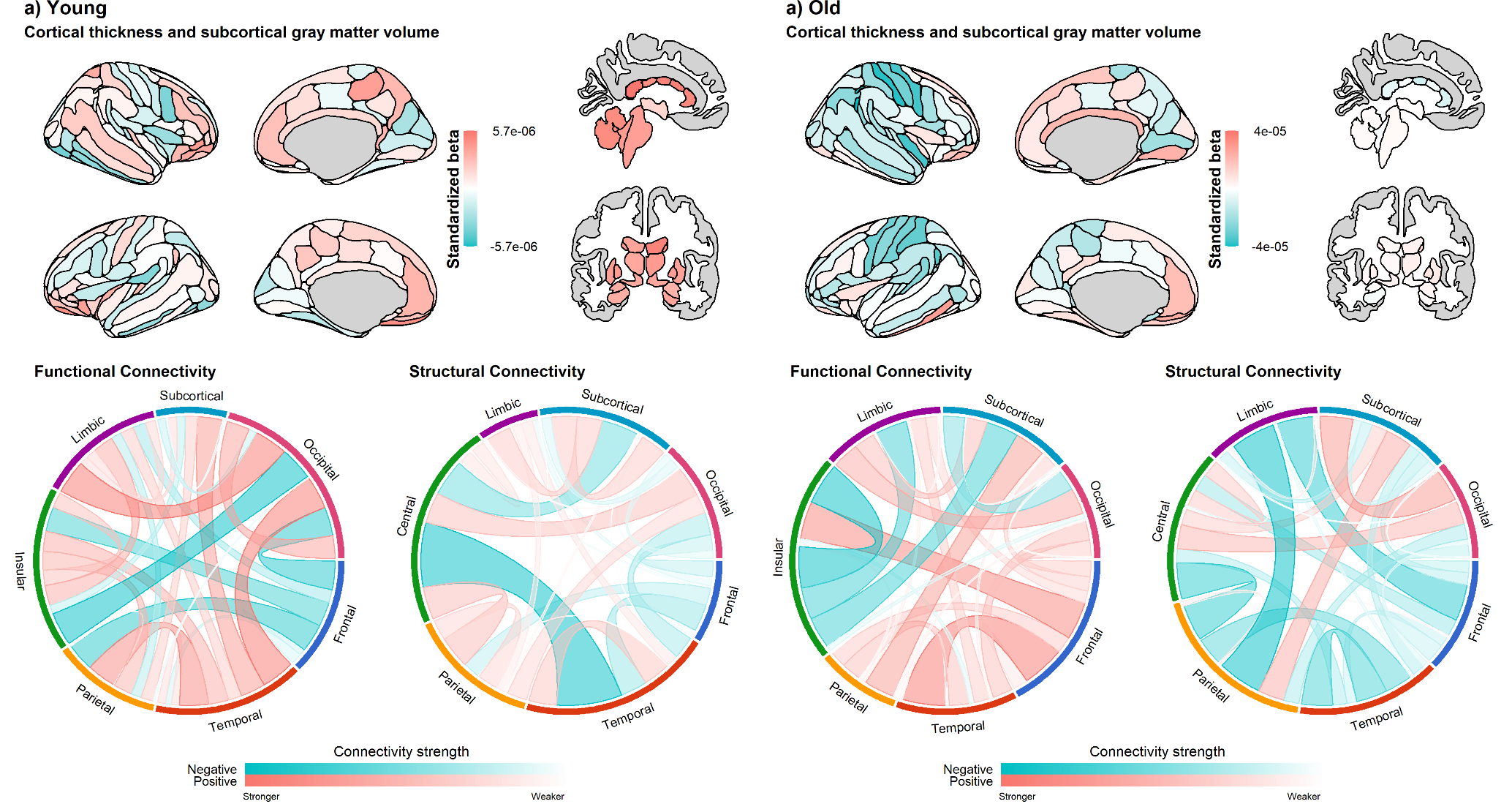
**

**Proverbs_Score**

**
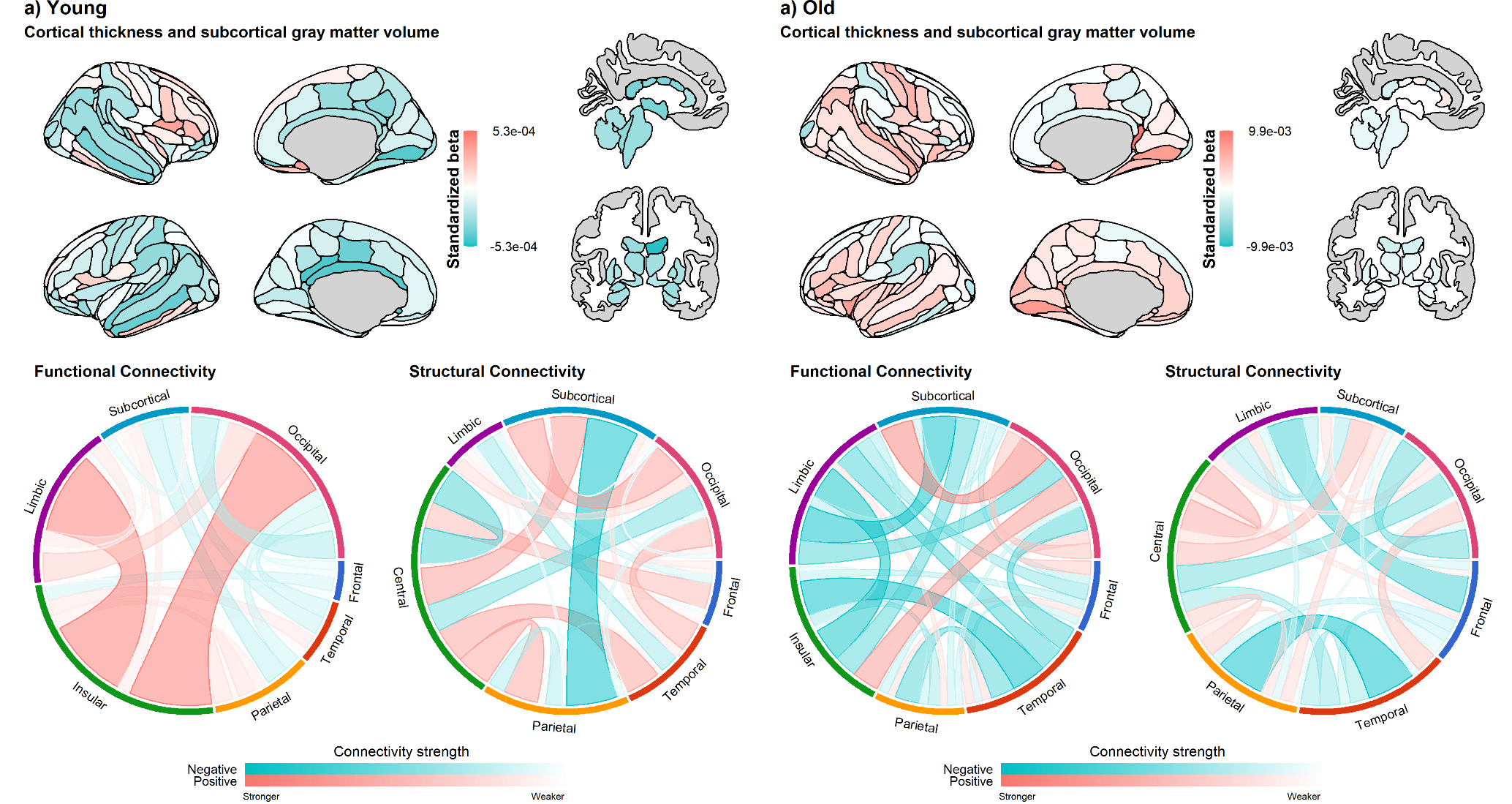
**

**RT_choice**

**
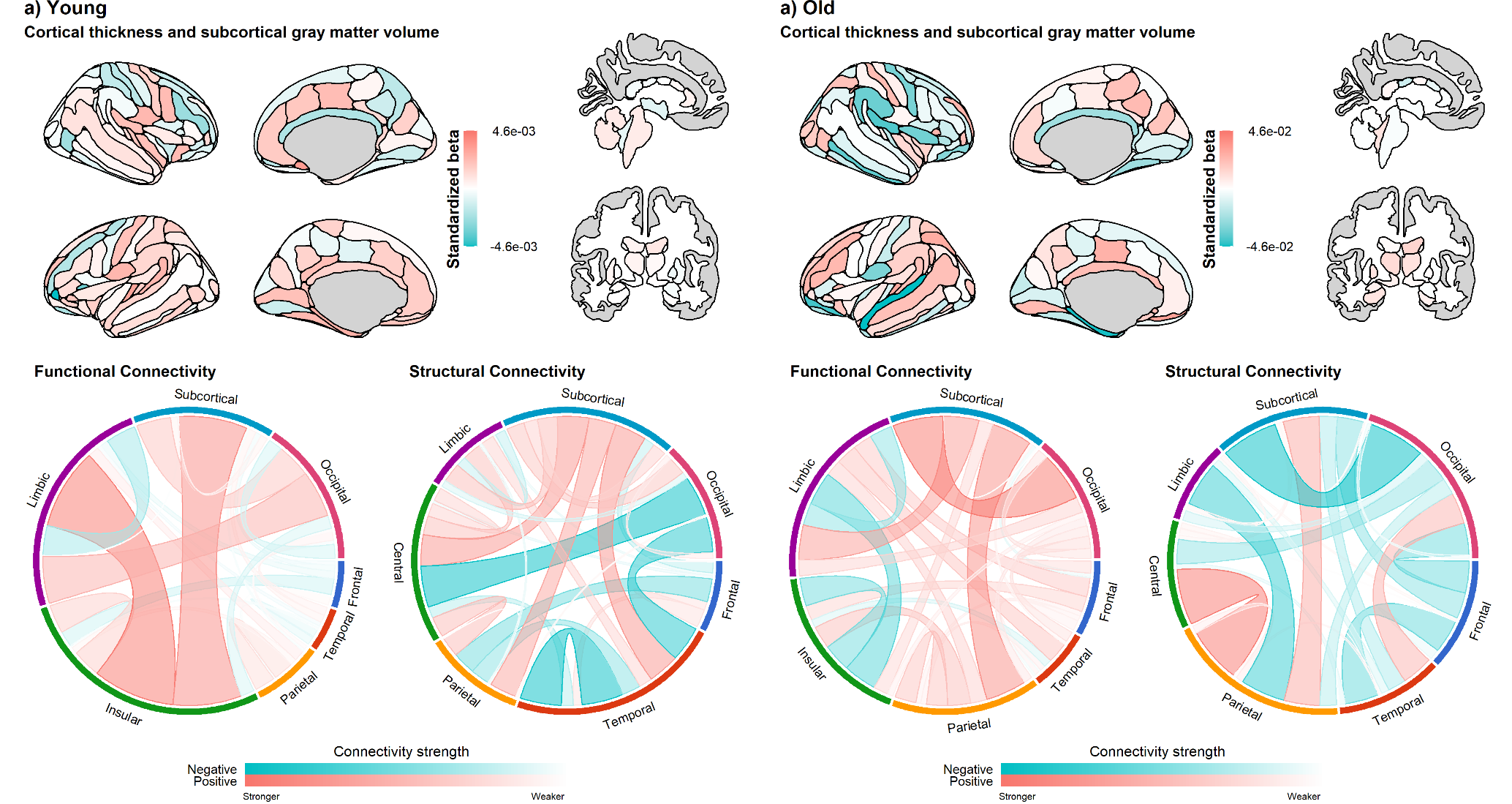
**

**Sensorimotor**

**
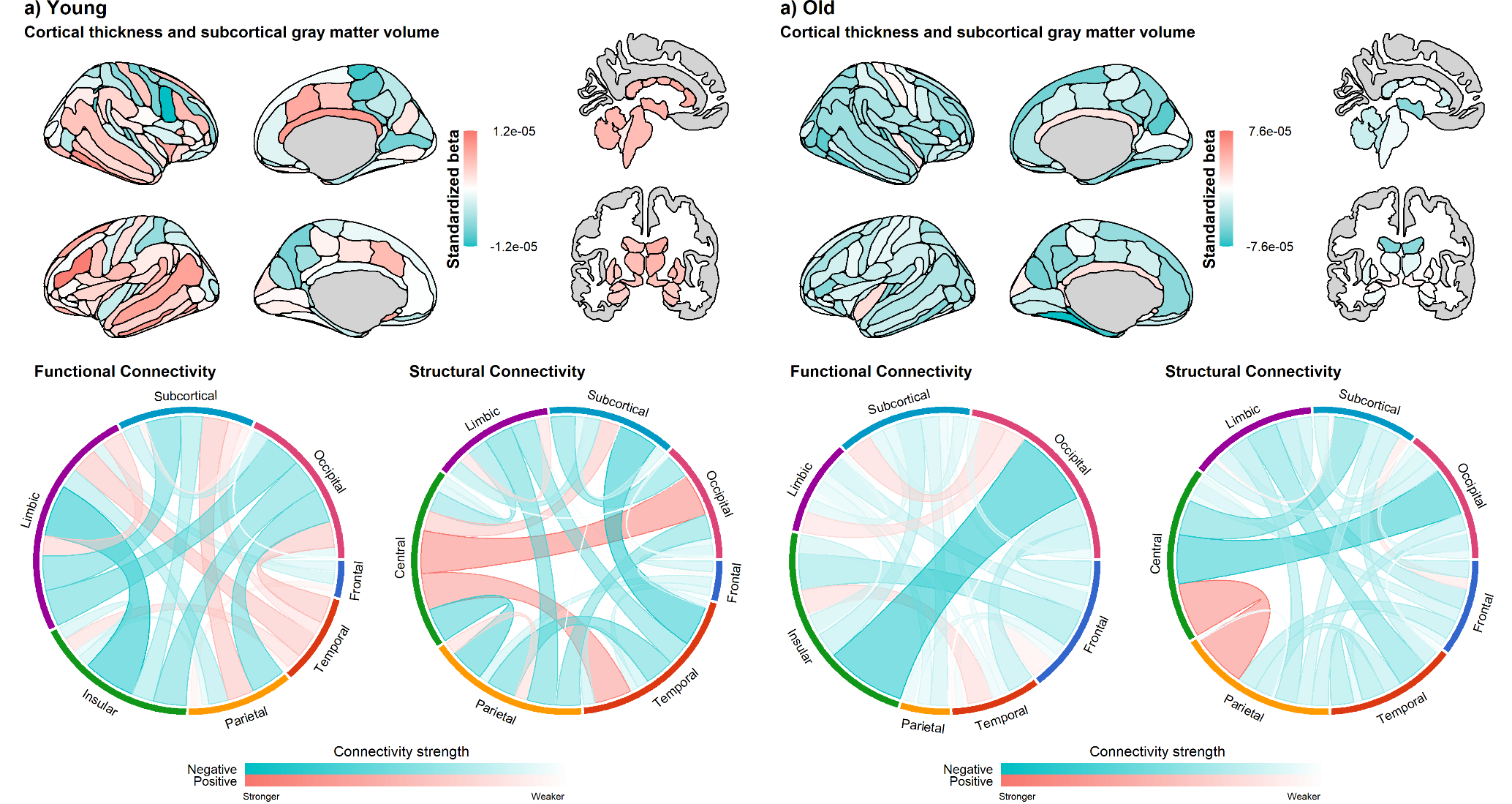
**

**VSTM**

**
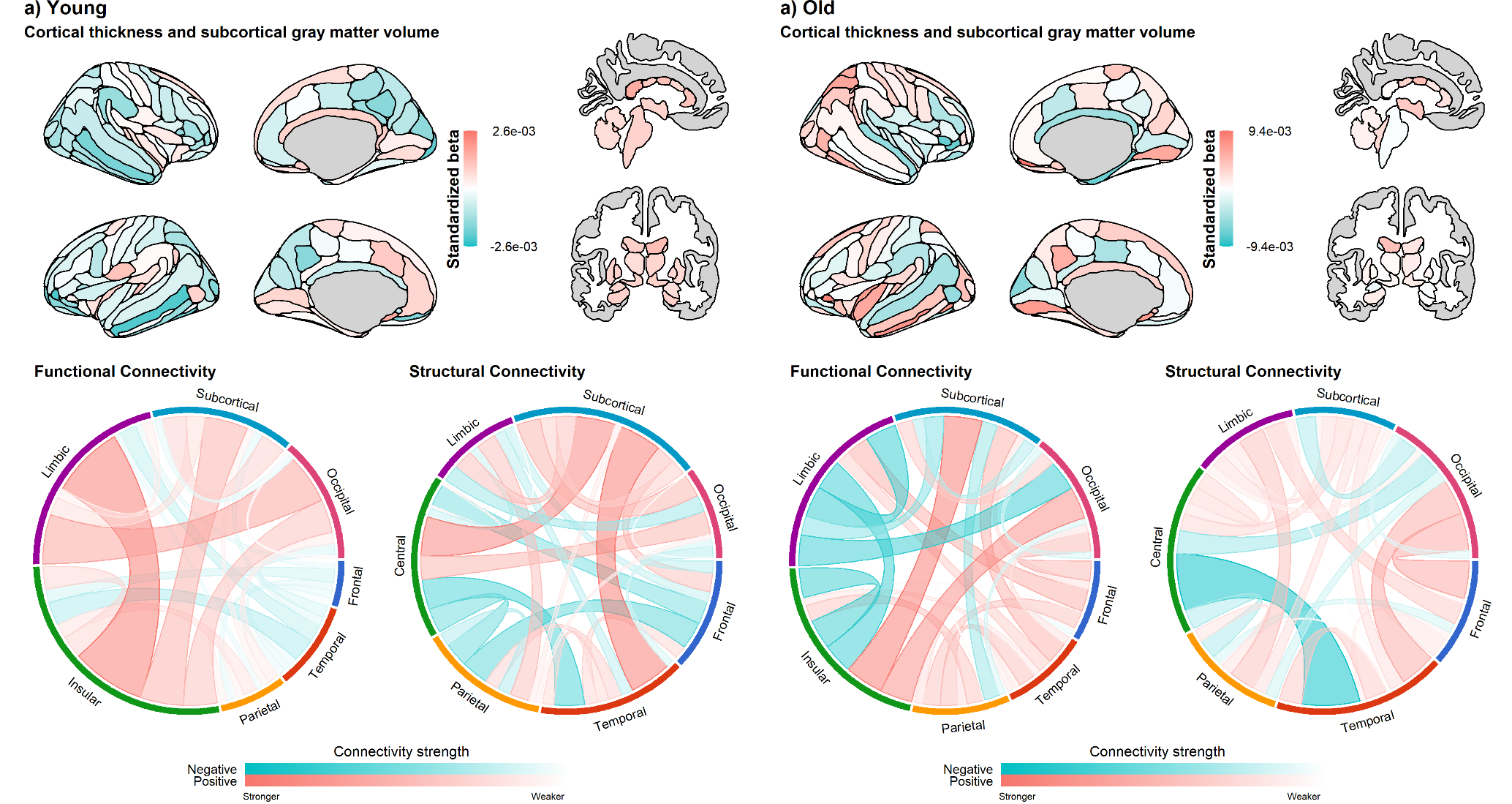
**
